# Anticancer Activity of the Acetylenic Derivative of Betulin Phosphate Involves Induction of Necrotic-Like Death in Breast Cancer Cells In Vitro

**DOI:** 10.3390/molecules26030615

**Published:** 2021-01-25

**Authors:** Arkadiusz Orchel, Ewa Chodurek, Marzena Jaworska-Kik, Piotr Paduszyński, Anna Kaps, Elwira Chrobak, Ewa Bębenek, Stanisław Boryczka, Paulina Borkowska, Janusz Kasperczyk

**Affiliations:** 1 Department of Biopharmacy, Faculty of Pharmaceutical Sciences in Sosnowiec, Medical University of Silesia, Katowice, Poland, 8 Jedności Str., 41-208 Sosnowiec, Poland; echodurek@sum.edu.pl (E.C.); jkik@sum.edu.pl (M.J.-K.); ppaduszynski@sum.edu.pl (P.P.); akaps@sum.edu.pl (A.K.); janusz.kasperczyk@sum.edu.pl (J.K.); 2 Department of Organic Chemistry, Faculty of Pharmaceutical Sciences in Sosnowiec, Medical University of Silesia, Katowice, Poland, 4 Jagiellońska Str., 41-200 Sosnowiec, Poland; echrobak@sum.edu.pl (E.C.); ebebenek@sum.edu.pl (E.B.); boryczka@sum.edu.pl (S.B.); 3 Department of Medical Genetics, Faculty of Pharmaceutical Sciences in Sosnowiec, Medical University of Silesia, Katowice, Poland, 8 Jedności Str., 41-208 Sosnowiec, Poland; pborkowska@sum.edu.pl

**Keywords:** betulin, cytotoxicity, apoptosis, regulated necrosis, breast cancer

## Abstract

Betulin (BT) is a natural pentacyclic lupane-type triterpene exhibiting anticancer activity. Betulin derivatives bearing propynoyloxy and phosphate groups were prepared in an effort to improve the availability and efficacy of the drug. In this study, a comparative assessment of the in vitro anticancer activity of betulin and its four derivatives was carried out using two human breast cancer cell lines: SK-BR-3 and MCF-7. In both studied cell lines, 30-diethoxyphosphoryl-28-propynoylbetulin (compound **4**) turned out to be the most powerful inhibitor of growth and inducer of cellular death. Detailed examination of that derivative pertained to the mechanisms underlying its anticancer action. Treatment with compound **4** decreased DNA synthesis and up-regulated p21^WAF1/Cip1^ mRNA and protein levels in both cell lines. On the other hand, that derivative caused a significant increase in cell death, as evidenced by increased lactate dehydrogenase (LDH) release and ethidium homodimer uptake. Shortly after the compound addition, an increased generation of reactive oxygen species and loss of mitochondrial membrane potential were detected. The activation of caspase-3 and fragmentation of genomic DNA suggested an apoptotic type of cell death. However, analysis of cellular morphology did not reveal any nuclear features typical of apoptosis. Despite necrosis-like morphology, dead cells exhibited activation of the cascade of caspases. These observations have led to the conclusion that compound **4** pushed cells to undergo a form of necrotic-like regulated cell demise.

## 1. Introduction

Pentacyclic lupane-type triterpenes are an interesting class of naturally occurring substances with a wide range of biological properties. Amidst them, betulin (BT) and betulinic acid (BA) represent two of the most extensively studied molecules, exhibiting broad anticancer activity among others [1]. Betulin (lup-20(29)-ene-3β,28-diol) is a triterpenoid widely distributed in plants that is particularly abundant in the outer bark of birch trees (up to 30% of dry weight) [2]. This raw material is readily available as a waste by-product in the wood industry, whereby BT can be extracted on a large scale. BT has been shown to exert cytotoxicity against some human neoplastic cell lines derived from cervical (HeLa), liver (HepG2, SK-HEP-1), lung (A549), and breast (MCF-7) cancers, as well as melanoma (G361), colorectal carcinoma (HCT116, HT29), and prostate tumor (PC-3) [3,4,5,6]. Interestingly, Rzeski et al. [1] reported that primary cultures of various neoplastic cells were more sensitive to BT treatment than established tumor cell lines. The anticancer activity of BA, a product of BT oxidation, has been proven in several studies, using both in vitro and animal models [7,8]. There is a growing body of evidence that the anticancer action of both substances is mediated mainly via apoptosis induction [9,10].

Programmed cell death, such as apoptosis, plays a crucial role in the maintenance of tissue homeostasis, ensuring a balance between cell proliferation and loss [11]. Apoptosis is manifested by characteristic morphological changes, such as cell shrinkage, condensation of chromatin, and nuclear fragmentation. Biochemical changes include the activation of a cascade of caspases, which are a group of cysteine proteases responsible for the execution of cellular death. Other biochemical modifications are internucleosomal DNA fragmentation, phosphatidylserine translocation to the outer leaflet of the plasma membrane, poly(ADP-ribose) polymerase (PARP) cleavage, protein cross-linking, and the peroxidation of membrane lipids [12,13]. Apoptotic cells retain their plasma membrane integrity relatively long, preventing the release of cytoplasmic contents into the surrounding tissue and induction of inflammatory response. There are two main pathways of apoptosis induction: extrinsic and intrinsic. The extrinsic pathway is initiated by the activation of death receptors by appropriate ligands, e.g., FasL, TNF-α, and Apo2L. Then, a death-inducing signaling complex is formed, resulting in the activation of caspase-8, which initiates the activation of the caspase cascade. Mitochondria play a crucial role in switching on the intrinsic apoptotic pathway. Proapoptotic stimuli cause a rapid increase in the permeability of the mitochondrial membrane, resulting in the release of proapoptotic factors: cytochrome c, Smac/DIABLO, HtrA2/Omi, AIF (apoptosis-inducing factor), and endonuclease G (EndoG). The first three proteins are involved in the activation of the caspase cascade, wherein caspase-9 and caspase-3 occupy key positions [14]. Active caspases cleave a variety of intracellular proteins, leading to their inactivation or activation. The best-known examples are PARP, nuclear lamins, inhibitor of CAD (ICAD), Bcl-2, DNA-dependent protein kinase, protein kinase Cδ, p21-activated kinase 2 (PAK2), gelsolin, and nuclear protein NuMA. On the other hand, AIF and EndoG function in a caspase-independent manner and can execute apoptotic changes in cells that lack caspase-3, such as the MCF-7 cell line [15]. Traditionally, necrotic cell death was considered as an uncontrolled and accidental process, which is extremely different from apoptosis. However, actually, this point of view seems to be obsolete, as there are numerous evidences that necrosis can be a strictly regulated process. In fact, a plethora of types of regulated necrosis has been described so far. They can be triggered by diverse pathophysiological stimuli and various intracellular mechanisms have been shown to mediate death signals. Among the best studied, such activating phenomena are mitochondrial permeability transition, PARP1 hyperactivation, inhibition of the Cys/Glu antiporter, necrosome (a protein complex containing activated RIPK1 and RIPK3 kinases) formation, as well as NAD^+^ and ATP depletion, oxidative stress, and Ca^2+^ overload [16]. The existence of an apoptosis–necrosis continuum, a biochemical network common for both apoptosis and necrosis processes, has been postulated [13]. Some factors, disturbing intracellular metabolic or signaling pathways, can shift an ongoing apoptotic process into necrosis [17]. On the other hand, numerous agents traditionally linked with necrosis can lead to an apoptotic phenotype [18]. A lot of stimuli have the ability to lead to a concomitant induction of apoptosis and necrotic-like cellular death in various populations of cells, both in vitro and in vivo [13,18,19].

Both betulin and betulinic acid seem to trigger the intrinsic pathway of apoptosis with cytochrome c release and subsequent caspase-9 and -3 activation [3,19]. Mitochondrial membrane permeabilization and reactive oxygen species generation were two events of crucial importance for cell death regulation. Cell pretreatment with blockers of the mitochondrial permeability transition pore or antioxidants suppressed apoptosis induced by BT and BA. The role of the Bcl-2 family of proteins in mitochondria permeabilization, in cells treated with BT and BA, remains unclear [10,19]. On the other hand, some authors [20,21] reported that BA induced an alternative caspase-independent cell death program in HeLa and Jurkat cell lines. However, precise molecular pathways as well as morphological features of that caspase-independent cell demise remain to be identified. Chemical modifications of the basic BT molecule led to the synthesis of several derivatives with enhanced cytotoxicity [22,23,24,25,26]. Some of them were demonstrated to induce cancer cell apoptosis via activation of the mitochondrial death pathway [27,28,29]. Therefore, it can be assumed that the disturbance of mitochondrial homoeostasis constitutes an important mechanism of anticancer activity of this whole class of substances.

**Figure 1:**
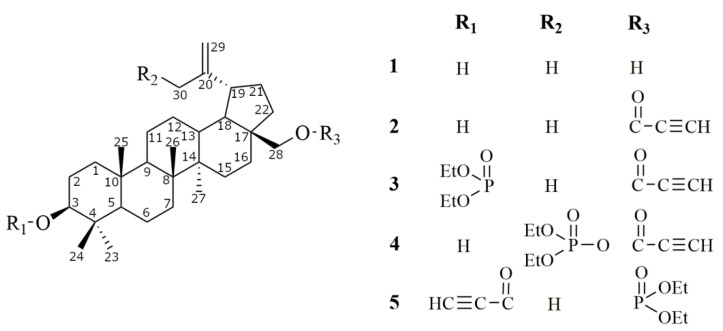
Chemical structure of betulin (**1**) and its four derivatives (**2**–**5**).

Previously, we have shown that substitution of the hydroxyl group at the C28 position of BT with a propynoyloxy function results in a significant augmentation of cytotoxicity [4,30]. Subsequently, we developed a series of betulin derivatives bearing both propynoyloxy and phosphate groups (Figure 1).

The introduction of a phosphate group to a drug substance molecule may be aimed at transforming it into a prodrug form, thus improving various properties, such as bioavailability and solubility [31]. On the other hand, the presence of a dialkylphosphate substituent may positively influence the pharmacological activity of the compounds and may even broaden the spectrum of biological activity of the obtained derivatives. Studies on dialkylphosphate derivatives of non-steroidal anti-inflammatory drugs, such as sulindac, aspirin, ibuprofen, and flurbiprofen, have shown their anticancer effects [32,33].

The aim of the present study was to evaluate the in vitro cytotoxic activity of several acetylenic derivatives of betulin and betulin phosphates toward two human breast cancer cell lines. Additionally, mechanisms underlying cytotoxic effects of the most active substance were analyzed in detail. Our studies included measurement of cell proliferation indices, p21^WAF1/Cip1^ gene expression, loss of mitochondrial membrane potential, oxidative stress, and plasma membrane integrity, as well as examination of cellular morphology and apoptotic response.

## 2. Results

### 2.1. Synthesis of Betulin Derivatives (***2***–***5***)

The synthesis of derivatives **2**–**4** as well as the first stage of the synthesis of compound 5 have been described in the earlier works of our team [25,34,35,36]. Due to the fact that in preliminary studies, derivatives with the phosphate group showed promising cytotoxicity, we decided to conduct a wider study. To assess the effect of substitutions in the betulin molecule on the activity of the tested derivatives, we additionally synthesized compound **5** (Figure 1. The chemical structure of compound **5** was confirmed by analysis by spectroscopic methods, and the results are included in the Supplementary Materials (Figures S1–S5).

### 2.2. Assessment of Cell Proliferation

A comparative assessment of the antiproliferative activity of betulin and its four derivatives was carried out on two breast cancer cell lines, using sulforhodamine B (SRB) staining. Cell density was determined after the 3-day incubation period; results were expressed as IC_50_ values (compound concentration causing 50% of the maximum inhibitory effect) and are listed in Table 1. Generally, SK-BR-3 cells seem to be more sensitive both to unmodified and modified betulin, as all the IC_50_ values were lower than for MCF-7 cells. Substitution of the C-28 hydroxyl group of the parent substance with a propynoyloxy function (compound **2**) improved the compound cytostatic efficacy in the SK-BR-3 cell line. However, the MCF-7 cell line proved to be highly resistant to 28-propynoylbetulin (**2**), and for that substance, it was not possible to calculate IC_50_. The introduction of a diethoxyphosphoryl group at the C3 position (compound **3**) led to slightly increased antiproliferative effects, but MCF-7 cells still remained substantially resistant. However, position replacement of these functional groups (compound **5**) yielded a molecule with a bit higher activity in relation to both cell lines compared to the original substance. 30-diethoxyphosphoryl-28-propynoylbetulin (**4**) demonstrated the strongest activity among studied betulins, achieving the lowest IC_50_ values.

**Figure 2:**
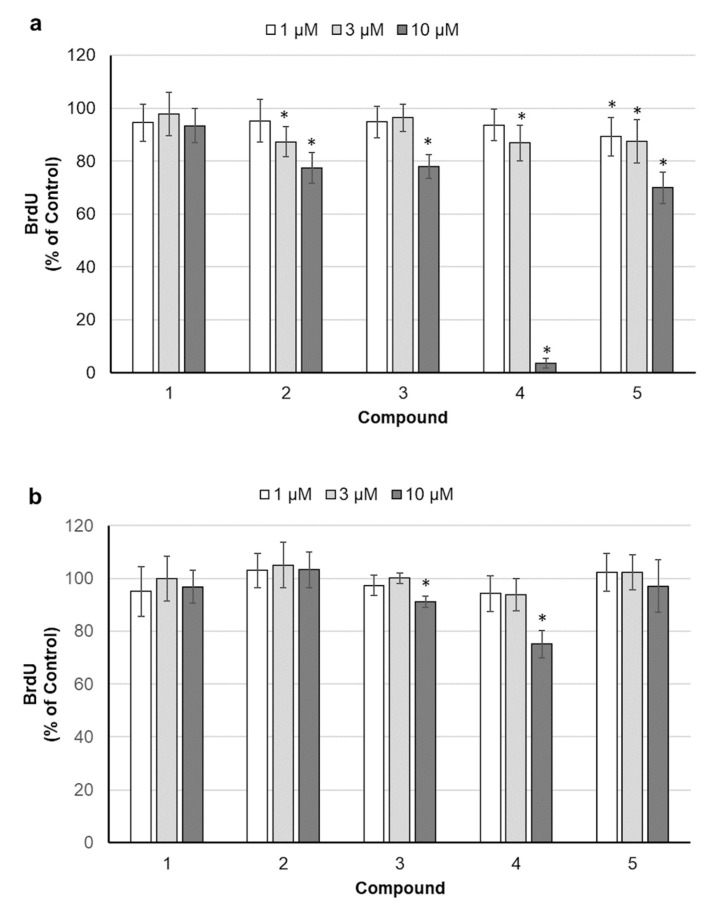
Effect of 24 h exposure to betulin and its derivatives on bromodeoxyuridine (BrdU) incorporation in SK-BR-3 (**a**) and MCF-7 (**b**) cells; the results are shown as mean ± SD; * *p* < 0.05 compared with control.

In order to define if the decreased cell density, revealed by SRB staining, was the consequence of reduced cell divisions, bromodeoxyuridine (BrdU) incorporation assay was carried out. BrdU, a structural analog of thymidine, is incorporated into the newly synthesized DNA during the S phase of the cell cycle. To identify substances capable of rapid blocking of the cell cycle, cells were exposed to betulins for a relatively short 24 h period, and the highest concentrations were eliminated to avoid acute toxicity. As shown in Figure 2, all betulin derivatives reduced somewhat the DNA synthesis rate in SK-BR-3 cells, with compound **4** being the most effective. MCF-7 cells turned out to be more resistant. In that cell line, only compounds **3** and **4** significantly reduced DNA synthesis at the highest concentration used.

### 2.3. Analysis of Cell Death

**Figure 3:**
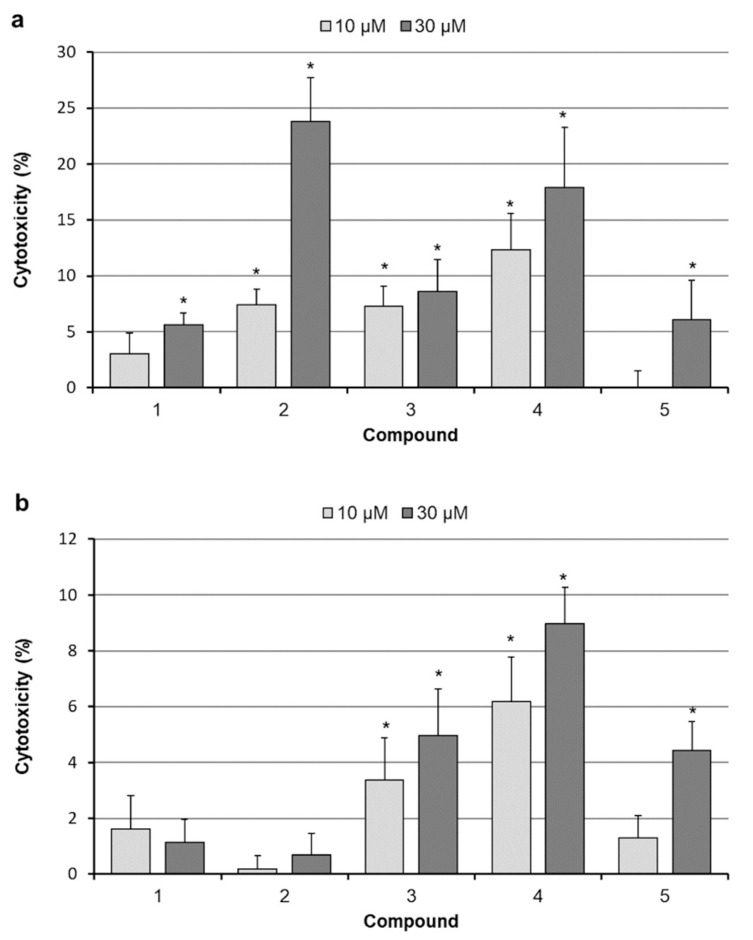
Effect of 24 h exposure to betulin and its derivatives on LDH release from SK-BR-3 (**a**) and MCF-7 (**b**) cells; the results are shown as percentage of maximal LDH release induced by cell lysis; each bar represents the mean ± SD; * *p* < 0.05 compared with control.

The leakage of LDH into culture medium was used as an indicator of cell death, as the last one results in loss of plasma membrane integrity. Based on the results of the above described studies, we chose betulins concentrations that are able to exert noticeable cytotoxic effects. As shown in Figure 3a, 24 h treatment with all drugs, except 10 µM compound **5**, caused some LDH release in SK-BR-3 cell cultures. The statistical analysis revealed significant differences (compared to control) in cultures treated with all compounds. Compounds **2**–**4** caused significant LDH release at both applied concentrations. It is worth noting that 28-propynoylbetulin (**2**) and 30-diethoxyphosphoryl-28-propynoylbetulin (**4**) were the most cytotoxic agents. Generally, MCF-7 cells response to the tested substances was markedly weaker, as amounts of the released enzyme were lower (Figure 3b). An interesting difference was seen in cell response to 28-propynoylbetulin (**2**). In SK-BR-3 cell cultures, it induced the highest rate of LDH release (23.8%), whereas MCF-7 cells appeared to be almost completely insensitive to that substance. The greatest loss of MCF-7 cells viability occurred after treatment with compound **4**.

**Figure 4:**
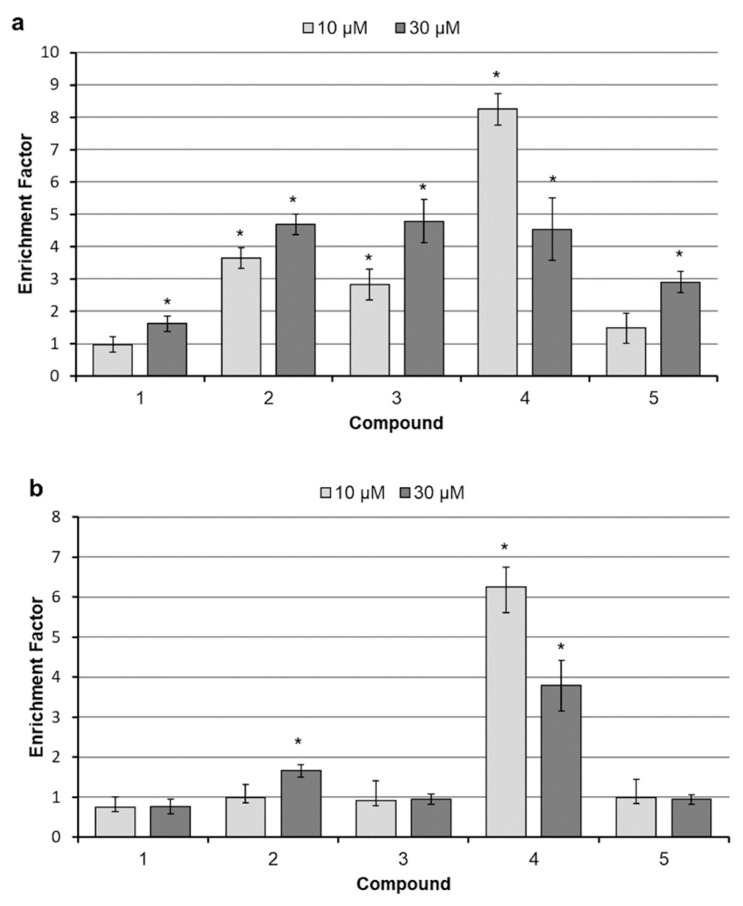
Determination of DNA fragmentation in SK-BR-3 (**a**) and MCF-7 (**b**) cells; cells were incubated with betulin and its derivatives for 24 h; cytosolic DNA/histone complexes were measured by ELISA; results are expressed as fold change over control; each bar represents the mean ± SD; * *p* < 0.05 compared with control.

In order to determine the relationship between reduced cell viability and activation of programmed cell death mechanisms, an effect of betulins on internucleosomal DNA fragmentation was analyzed. As shown in Figure 4a, treatment for 24 h with all studied compounds induced significant DNA fragmentation in the SK-BR-3 cell line. Compounds **1** and **5** caused moderate increases only at the higher concentration. The remaining compounds led to significant chromatin fragmentation at both applied concentrations. Interestingly, DNA degradation caused by the compound **4** seems to be much stronger at lower 10 µM concentration compared with 30 µM. Presumably, it is a consequence of the rapid progression of necrotic phenomena, including disintegration of the cell membrane. Most probably, the increased permeability of the cell membranes resulted in the release of DNA fragments to culture medium, making them inaccessible for the assay. MCF-7 cells were again more resistant to betulins. Moderate DNA fragmentation resulted from treatment with 30 µM compound **2** (Figure 4b). Substance **4** induced considerable disintegration of the genetic material, especially at lower 10 µM concentration.

### 2.4. 30-diethoxyphosphoryl-28-propynoylbetulin Induces P21^WAF1/Cip1^ mRNA and Protein Expression

**Figure 5:**
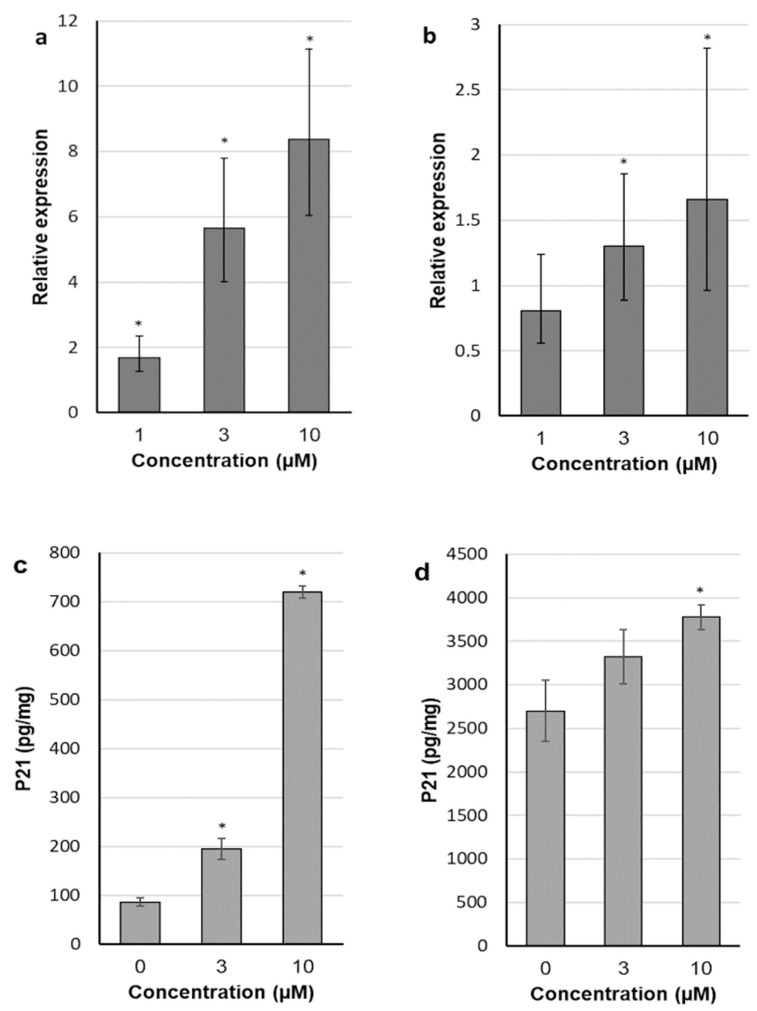
Effect of 6 h exposure to betulin and its derivatives on p21 mRNA (**a**,**b**) and protein (**c**,**d**) expression in SK-BR-3 (**a**,**c**) and MCF-7 (**b**,**d**) cell lines; each bar represents the mean ± SE (**a**,**b**) or SD (**c**,**d**); mRNA levels are expressed as fold change over control; * *p* < 0.05 compared with control.

In view of the above described results from comparative studies, it appears that introduction of the diethyl phosphate group at the C-30 position of betulin and the propynoyl group at the C-28 position resulted in substance (**4**) able to efficiently inhibit proliferation and induce cell death in two breast cancer cell lines. Therefore, 30-diethoxyphosphoryl-28-propynoylbetulin (**4**) was selected to the in-depth research of mechanisms underlying its anticancer activity. First, the effect of compound **4** on P21^WAF1/Cip1^ expression, an important regulator of the cell cycle progression, was studied. As shown in Figure 5a,b, compound **4** in a dose-dependent manner increased the P21^WAF1/Cip1^ mRNA level, relative to control cultures (expression in control is equivalent to 1). In SK-BR-3 cells treated with concentration of 10 µM, transcript amount was increased even up to 8.37-fold, compared to control. In MCF-7 cells, the P21^WAF1/Cip1^ transcript level was significantly elevated after treatment with the derivative **4** at concentrations of 3 and 10 µM. The results presented in Figure 5c,d show that P21 protein concentrations roughly followed the same pattern as P21^WAF1/Cip1^ transcript levels. Generally, MCF-7 cells presented more modest responses to the tested substance both at the mRNA and protein level.

### 2.5. 30-diethoxyphosphoryl-28-propynoylbetulin Causes an Early Damage of Mitochondrial Function

**Figure 6:**
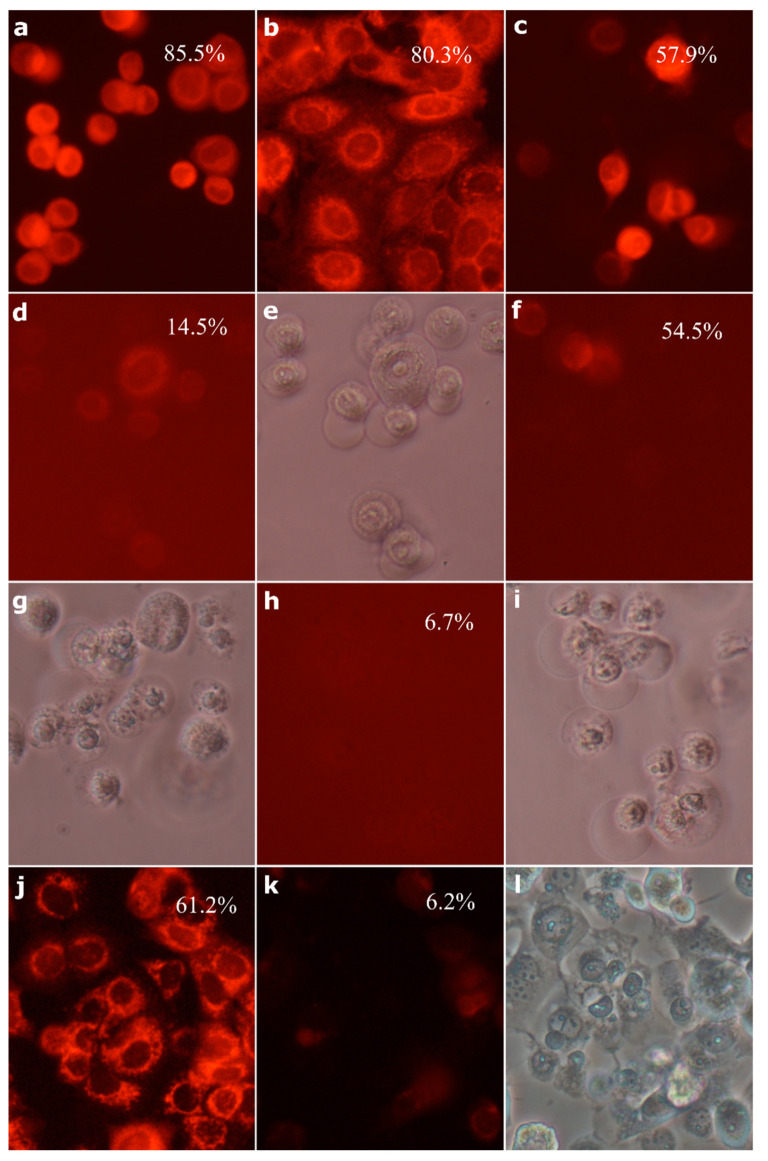
Effect of compound **4** on the mitochondrial membrane potential: control SK-BR-3 cells (**a**); control MCF-7 cells (**b**); SK-BR-3 cells treated with 10 µM compound **4** for 3 h (**c**); SK-BR-3 cells treated with 30 µM compound **4** for 3 h (**d**); SK-BR-3 cells treated with 10 µM compound **4** for 6 h (**f**); SK-BR-3 cells treated with 30 µM compound **4** for 6 h (**h**); MCF-7 cells treated with 10 µM compound **4** for 6 h (**j**); MCF-7 cells treated with 30 µM compound **4** for 6 h (**k**); numbers indicate percentage of TMRM positive cells determined by flow cytometry; phase-contrast photomicrograph of cells treated with compound **4**: SK-BR-3 cells treated with 30 µM compound **4** for 3 h (**e**); SK-BR-3 cells treated with 10 µM compound **4** for 6 h (**g**); SK-BR-3 cells treated with 30 µM compound **4** for 6 h (**i**); MCF-7 cells treated with 30 µM compound **4** for 6 h (**l**).

Tetramethylrhodamine methyl ester (TMRM) is a cationic fluorescent stain, which accumulates in mitochondria proportionally to their negative transmembrane potential. The incubation of cells with healthy mitochondria with TMRM results in their strong red–orange fluorescence (Figure 6a,b). As shown in Figure 6c,d,f,h,j,k, the incubation of SK-BR-3 cells with compound **4** resulted in the rapid disappearance of red fluorescence, especially in cells treated with a 30 µM dose. As early as 3 h after exposure to the compound, mitochondria were depolarized in a substantial number of cells. Cell treatment with the higher concentration for 6 h resulted in a dissipation of mitochondrial membrane potential in almost all cells. Generally, MCF-7 cells seemed somewhat more resistant, as 3 h treatment with 10 µM compound **4** was not sufficient to depolarize mitochondria in that cell line (data not shown).

### 2.6. 30-diethoxyphosphoryl-28-propynoylbetulin Increases Caspase-3 Activity

**Figure 7:**
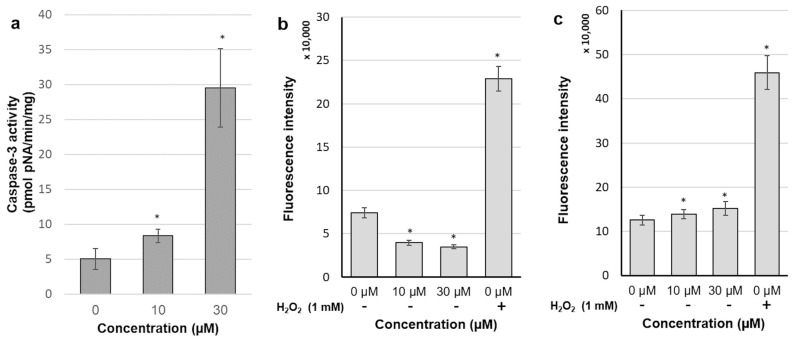
Effect of 6 h exposure to compound **4** on caspase-3 activity in the SK-BR-3 cell line (**a**); effect of compound **4** on ROS generation in SK-BR-3 cells (**b**); effect of compound **4** on ROS generation in the MCF-7 cell line (**c**); each bar represents the mean ± SD; * *p* < 0.05 compared with control.

As the MCF-7 cell line lacks a functional caspase-3 protein, this study was done on SK-BR-3 cells only. Compound **4** significantly increased enzyme activity, especially at a concentration of 30 µM (Figure 7a), which enlarged the studied parameter by six-fold. This observation suggested again the strong proapoptotic activity of the tested substance.

### 2.7. Intracellular Production of Reactive Oxidative Species

DCFDA, a redox-sensitive fluorescent probe, is easily taken up by human cells and deacetylated by cellular esterases. The resulting 2′,7′-dichlorodihydrofluorescein is retained intracellularly and oxidized by ROS into 2′,7′-dichlorofluorescein (DCF), which is a highly fluorescent substance. Treatment of both our cell lines with 1 mM H_2_O_2_ (positive control) resulted in a strong induction of DCF fluorescence. However, 30 min incubation with compound **4** exerted quite diverse effects on ROS production in each cell line. As shown in Figure 7c, both doses of compound **4** caused the slight but statistically significant increase in ROS production in MCF-7 cells. A rapid induction of free radicals formation could be grounds for the subsequent depolarization of mitochondria mediated via activation of the mitochondrial permeability transition pore (MPTP). Surprisingly, free radicals levels in SK-BR-3 cells were substantially reduced after incubation with the tested substance (Figure 7b). The same pattern of ROS production was noticed at various periods of incubation with compound **4**, ranging from 15 min to 3 h.

### 2.8. Morphological Analysis of Cells Treated with 30-diethoxyphosphoryl-28-propynoylbetulin

**Figure 8:**
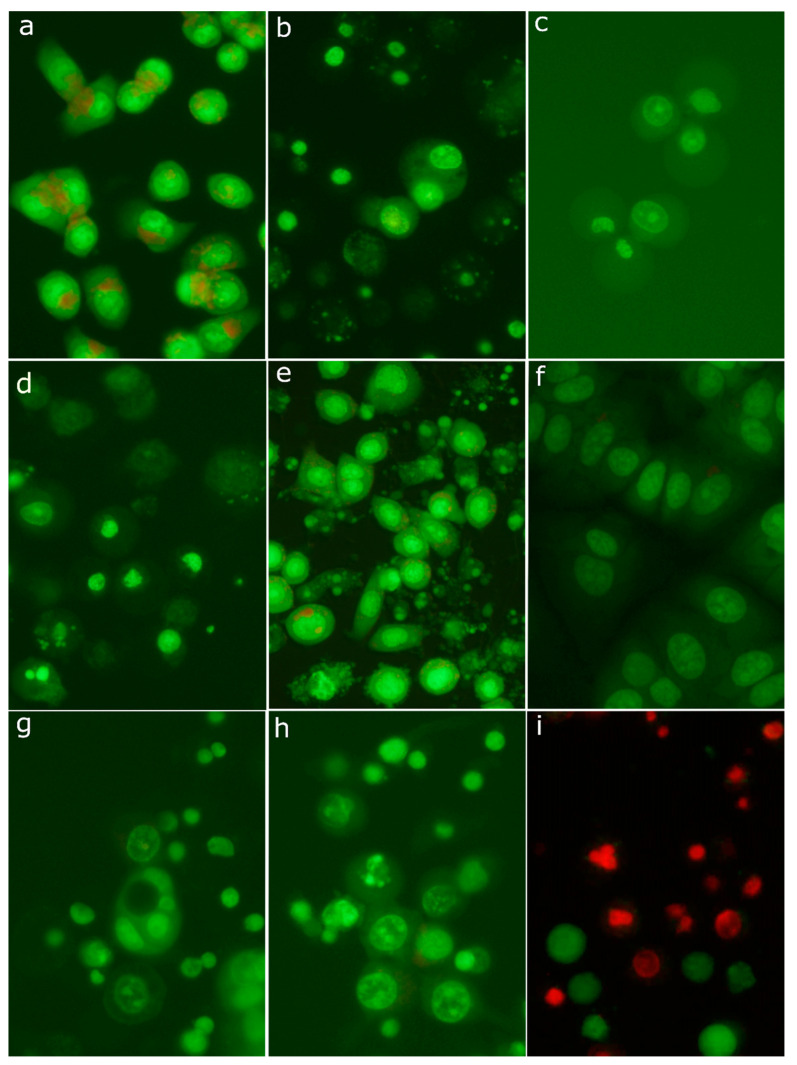
Morphology of SK-BR-3 cells stained with acridine orange: control cells (**a**); cells treated with 10 µM compound **4** for 24 h (**b**); cells treated with 30 µM compound **4** for 6 h (**c**); cells treated with 30 µM compound **4** for 24 h (**d**); cells treated with 20 mM sodium butyrate (positive control) (**e**); morphology of MCF-7 cells stained with acridine orange: control cells (**f**); cells treated with 10 µM compound **4** for 24 h (**g**); cells treated with 30 µM compound **4** for 24 h (**h**); effect of compound **4** (30 µM, 24 h) on SK-BR-3 cell viability assessed using the Live/Dead Viability/Cytotoxicity Kit (**i**).

Taking into account increased apoptotic indices (DNA fragmentation, caspase-3 activity), we evaluated in detail morphological changes of cell nuclei. Analysis of cell morphology is one of the most important methods to identify apoptotic cell death. The use of fluorescent DNA intercalating dyes, such as acridine orange, enables visualization of the cell nucleus and observation of morphological changes characteristic of apoptosis. Untreated SK-BR-3 and MCF-7 cells stained with acridine orange presented mainly interphase nuclei with relaxed chromatin (Figure 8a,f). Additionally, acid vesicles such as lysosomes with red fluorescence were visible. As shown in Figure 8e, treatment of the SK-BR-3 cell line with 20 mM sodium butyrate (positive control) induced typical morphological features of apoptosis, including chromatin condensation, nuclear shrinkage, and ultimately fragmentation of the nucleus into highly packed spherical parts. Finally, late apoptotic cells disintegrated into numerous fragments. Surprisingly, treatment with cytotoxic concentrations of compound **4** yielded very low number of cells with the apoptotic morphology. As can be seen in Figure 6e,g,i,l, treated cells detached from a substratum quickly become swollen, and big, transparent bubbles were formed, suggesting a necrotic mode of cell death.

**Figure 9:**
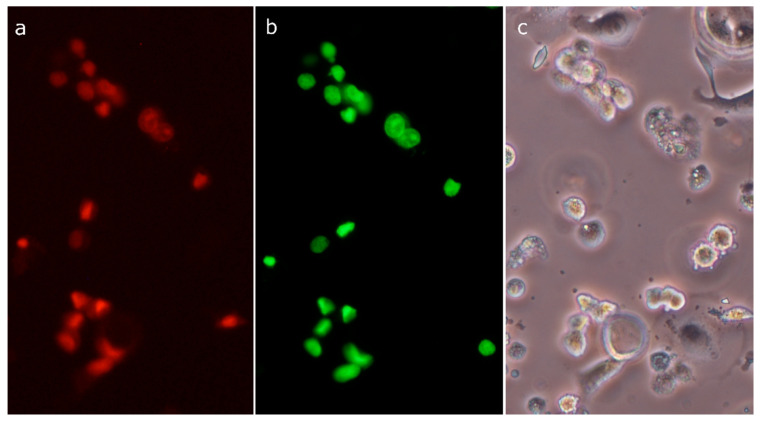
Imaging of active caspases in SK-BR-3 cell line treated with 30 µM compound **4** for 6 h, using an Image-iT™ LIVE Red Poly Caspases Detection Kit: active caspases visualized using FLICA reagent (FAM-VAD-FMK poly caspases reagent) (**a**); dead cells permeable to SYTOX Green nucleic acid stain (**b**); the same area as in the previous photographs seen in phase-contrast microscopy (**c**).

Nuclei of SK-BR-3 cells stained with acridine orange after 6 h treatment with 30 µM compound **4** presented two variants of morphological changes: some of them were distended and weakly stained, whereas others were rather shrunken and condensed (Figure 8c). After a 24 h treatment period, distended nuclei seemed almost completely degraded, whereas condensed nuclei appeared largely unchanged. In numerous cells, green clumps scattered in the cytoplasm were visible (Figure 8d). The morphology of some cells with these clumps somewhat resembled apoptosis. Staining of cells with the Live/Dead Viability/Cytotoxicity Kit revealed the same kinds of morphological changes in dead cells absorbing ethidium homodimer-1 (Figure 8i). However, there were no clumps in the cytoplasm or clumps exhibiting green fluorescence (originating from calcein). It means that clumps did not contain nuclear chromatin, but they were composed of cytoplasmic structures (e.g., clusters of mitochondria). Treatment with 10 µM compound **4** resulted in similar morphological changes, but their rate was much lower (Figure 8b). The MCF-7 cell line responded clearly slower to compound **4**, but after the 24 h treatment period, cells become detached and swollen with distended, weakly stained nuclei (Figure 8g,h). The above described observations of cellular morphology indicate a necrotic rather than apoptotic form of cell death. The labeling of treated SK-BR-3 cells with SR-VAD-FMK, the broad-spectrum fluorescent caspase inhibitor, revealed the extensive activation of caspases. After treatment with 30 µM compound **4** for 6 h, the majority of cells were positively labeled (Figure 9a). Simultaneously, cells demonstrated green fluorescence of SYTOX Green, indicating diminished viability and a leaky cellular membrane (Figure 9b). Most importantly, active caspases were detected in numerous floating cells without apoptotic morphology of the nucleus. Frequently, these cells demonstrated distended nuclei with loose chromatin. These observations evidence that in numerous SK-BR-3 cells, activation of the cascade of caspases preceded necrotic-like cell death. Only occasional staining (usually connected with the apoptotic morphology) was seen in control cultures. The above-described observations suggest that, in breast cancer cell lines, 30-diethoxyphosphoryl-28-propynoylbetulin induced some form of regulated necrosis.

## 3. Discussion

Breast cancer is one of the most common invasive cancers all over the world. The causes of breast cancer are not yet fully known, although a number of risk factors have been identified. The International Agency for Research on Cancer of the World Health Organization presented a report based on the GLOBOCAN (The Global Cancer Observatory) database estimating breast cancer occurrence on 2.1 million new cases and 626,000 deaths worldwide in 2018. In female patients, it is the most frequently diagnosed cancer in the majority of evaluated countries and also the leading cause of cancer-related death in over 100 countries [37,38]. The main factors related with increased risk of female breast cancer are especially: older age, inherited genetic mutations for breast cancer (BRCA1 and/or BRCA2), atypical hyperplasia history, high endogenous estrogen or testosterone levels, mammographically dense breast, family history of cancer, tall adult height, early menarche and late menopause, late age at first full-term pregnancy, obesity, recent and long-term use of menopausal hormone therapy containing estrogen and progestin, never breastfeeding, high bone mineral density in postmenopausal women, diethylstilbestrol exposure, and consumption of alcohol [39,40,41,42,43].

One of the most characteristic features of the cancer cell is resistance to apoptosis, determining both tumor ability to grow and resistance to treatment. Diminished apoptosis in breast cancer often results from dysregulation of the BCL-2 family of proteins, which regulates the permeability of the mitochondrial outer membrane [44]. For example, the Mcl-1 protein is frequently overexpressed in breast tumors, and it has been found to correlate with a bad prognosis [45]. On the other hand, the induction of apoptosis is a pivotal mechanism of action of numerous anticancer agents, determining the effectiveness of therapy. There are several reports describing the modifications of betulin molecule, resulting in derivatives with pronounced apoptotic activity. 3,28-di-(2-nitroxy-acetyl)-oxy-betulin induced apoptosis of the hepatocellular carcinoma Huh7 cell line, as a consequence of the mitochondrial pathway activation [46]. Betulin esters containing lysine and ornithine side chains (at C-28) efficiently induced apoptosis in the epidermoid squamous carcinoma A-431 cell line [47], as well as the melanoma Me-45 cell line [24]. Majeed et al. [48] found out that treatment of both leukemia and breast cancer cells with 3(1N(5-hydroxy-naphth-1yl)-1H-1,2,3-triazol-4yl)methyloxy betulinic acid resulted in the growth arrest and apoptosis. Cell death was proceeded by inhibition of the phosphatidylinositol-3 kinase pathway, decrease in the ratio of Bcl-2/Bax expression, generation of reactive oxygen species (ROS), dissipation of the mitochondrial membrane potential, and ultimately activation of the cascade of caspases. Introduction of a propynoyl motif at the C-28 position of betulin molecule enhanced its proapoptotic activity against the endometrial adenocarcinoma Ishikawa cell line [29] and malignant melanoma G-361 cells [4].

In this study, we found that 28-propynoylbetulin quite efficiently induced growth arrest and cell death in the SK-BR-3 cell line, but MCF-7 cells were substantially insensitive to that substance. A variable susceptibility of cells to that compound was reported previously [49] with T47D and MCF-7 breast cancer cell lines relatively insensitive. However, the insertion of an additional phosphate group at the C-30 position resulted in the compound displaying augmented cytotoxic activity against both cell lines used in our study. On one hand, reduced DNA synthesis and increased P21 gene expression suggest that impaired cell growth could be a result of blockade of the cell cycle and stopped cell divisions. On the other hand, increased LDH release and ethidium homodimer uptake indicate substantial cell death induction. Genomic DNA fragmentation and caspase-3 activation imply apoptosis as a predominant mode of cell death. Surprisingly, cells with typical apoptotic morphology were almost absent in cultures treated with cytotoxic concentrations of 30-diethoxyphosphoryl-28-propynoylbetulin. Rello et al. [50] demonstrated that cells subjected to necrotic treatments quickly developed numerous plasma membrane evaginations due to the loss of control of water influx through the plasma membrane. Then, these evaginations merged, forming a big, single bubble that ultimately detached from the cell surface. Cell nuclei underwent a uniform condensation of chromatin, leading to shrunken pyknotic nuclei. Soriano et al. [51] reported two types of necrotic nuclear morphology after the exposure of HeLa cells to photodynamic treatment. Some cells showed the small nucleus with highly and rather uniformly condensed chromatin, whereas other cells presented “spotted” nuclei with condensed chromatin granules. Other authors found that cell necrosis led to the swelling of cell nuclei [52,53,54]. Similarly, Pseudomonas aeruginosa-induced oncosis was characterized by swollen nuclei with dispersed chromatin and cell disintegration [55]. In this study, treatment of breast cancer cells with 30-diethoxyphosphoryl-28-propynoylbetulin caused morphological changes typical for necrotic stimuli. We observed cell swelling, the formation of giant vesicles from the plasma membrane, uniform condensation of chromatin, or contrary swelling of cell nuclei followed by their disintegration. These phenomena were preceded by collapse of the mitochondrial membrane potential and activation of caspase-3. Mitochondrial dysfunctions, such as the mitochondrial membrane permeability transition (MPT), disruption of the mitochondrial transmembrane potential, and cessation of oxidative phosphorylation have been identified as common events occurring at the early stage of both apoptotic and necrotic cell demise [56,57]. The ultimate mode of cell death seems to be determined by intracellular ATP level [17,58]. ATP depletion guides cells to necrotic death in response to signals inducing apoptosis at higher energy supply. Apoptosis includes several energy-dependent steps such as caspase-9 activation, active nuclear transport and chromatin condensation [59,60,61]. In some experimental models, the inactivation of caspases resulted in a shift from apoptotic to necrotic-like mode of cell death, which is characterized by the lack of nuclear apoptotic events [56,62]. Interestingly, cell necrosis is an inevitable consequence of the fully progressed apoptotic program if there are not phagocytic cells in the neighborhood. This stage is termed “secondary necrosis” and is characterized by low ATP content and permeable cell membrane, which can result in the release of cellular content, including DNA fragments, to the extracellular environment [31,36].

Experiments in mouse models revealed that activated caspase-3 is necessary for chromatin condensation, DNA fragmentation, and nuclear breakdown [63]. On the other hand, there are several reports showing that the MCF-7 cell line, lacking functional caspase-3, exhibited some aspects of nuclear apoptosis [64,65,66]. These publications remain in agreement with our observations where compound **4** caused significant DNA degradation in MCF-7 cells. Alternative mechanisms involving caspases-6/7 and AIF have been suggested to operate in these cells. However, a cascade of caspases activation is not sufficient to execute apoptosis. Some physiological processes, such as erythrocyte, monocyte or lens fiber cell terminal differentiation, as well as the stimulation and proliferation of T lymphocytes, have been demonstrated to involve turning on caspases [67,68]. Concomitantly, in some studies, caspases were activated and necessary for the induction of necrotic-like cell death [17,18,69]. It has been postulated that a sudden and massive permeability transition impairing substantially cellular metabolism before enzymatic digestion of appropriate nuclear and cytoplasmic substrates results in cell necrosis. If mitochondrial failure occurs in the more sluggish mode and the activation of proper enzymes takes place before ATP depletion, apoptosis can ensue [56]. Oxidative stress is a well-known factor that is able to induce apoptosis through the mitochondrial pathway [69]. Mitochondria contain numerous targets of ROS, and their oxidative modifications can trigger MPTP opening, organelle osmotic swelling, rupture of the outer membrane, and the release of cytochrome c. It has been shown that betulin induced an apoptosis of Jurkat cells involved in ROS generation [10]. Similarly, betulinic acid caused disruption of the mitochondrial potential followed by the hyperproduction of ROS in the neuroblastoma SH-EP cell line [9]. In the present study, an increased level of oxidative stress was detected in MCF-7 cells as early as 15 min after the compound **4** addition. It means that enhanced ROS generation occurred before depolarization of the mitochondrial membrane. The latter was noticeable approximately at 2 h incubation. Therefore, the observed increase in DCFDA oxidation could not be the result of a direct reaction with cytochrome c released from mitochondria to cytoplasm [70]. ROS are considered important intracellular signaling mediators that are involved in the regulation of various processes including cell cycle progression, cell motility, and induction of cell death [71,72]. Surprisingly, compound **4** rapidly decreased DCFDA oxidation in the SK-BR-3 cell line, suggesting lowered ROS generation, although in some studies, a reduction of ROS production preceding apoptosis was observed [72,73]. It has been postulated that the alteration of ROS level, whether an increase or decrease, may lead to activation of the stress response that ultimately ends in cell death.

## 4. Materials and Methods

### 4.1. Materials

**Figure 10:**
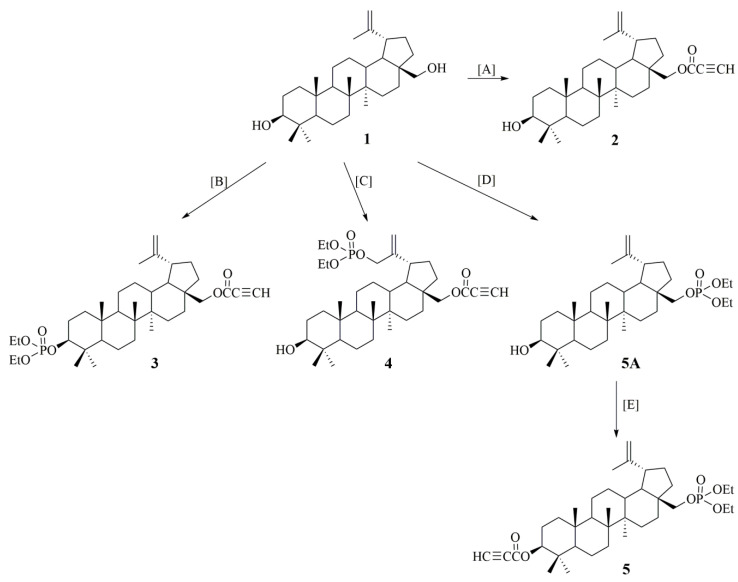
Synthesis pathways of betulin derivatives (**2**–**5**), used reagents and reaction conditions. A: propiolic acid, dicyclohexylcarbodiimide (DCC), 4-dimethylaminopyridine (DMAP), CH_2_Cl_2_, −10 °C—r. t. [25]; B: (i) acetic anhydride {(CH_3_CO)_2_O}, pyridine, CH_2_Cl_2_, r. t. (ii) diethylchlorophosphate {(EtO)_2_P(O)Cl}, DMAP, pyridine, r. t. (iii) NaOH, tetrahydrofuran (THF), H_2_O, methanol, r. t. (iv) propiolic acid, DCC, DMAP, CH_2_Cl_2_, −10 °C—r. t. [35]; C: (i) (CH_3_CO)_2_O, pyridine, CH_2_Cl_2_, r. t. (ii) m-chloroperbenzoic acid (m-CPBA), CHCl_3_, reflux. (iii) (EtO)_2_P(O)Cl, DMAP, pyridine, r. t. (iv) NaOH, THF, H_2_O, methanol, r. t. (v) propiolic acid, DCC, DMAP, CH_2_Cl_2_, −10 °C—r. t. [34] D: (EtO)_2_P(O)Cl, DMAP, THF, r. t. [36]; E: propiolic acid, DCC, DMAP, CH_2_Cl_2_, −10 °C—r. t.

Betulin (**1**) was purchased from Sigma Aldrich (Poznań, Polska) (98% purity). All betulin derivatives were synthesized in Department of Organic Chemistry, Faculty of Pharmaceutical Sciences in Sosnowiec, SUM. Structures of both the parent compound and derivatives are summarized in Figure 1. Compound **2** was a result of reaction of betulin with propynoic acid. To obtain the phosphate betulins (compounds **3**–**5**), respective monoacetate derivatives of betulin or 3,28-O,O-diacetyl-30-hydroxybetulin were used, concomitantly with diethyl chlorophosphate. Products, after the hydrolysis of protective acetyl groups, were reacted with propynoic acid to obtain respective acetylenic derivatives (Figure 10). Crude reaction products were purified by the gel column chromatography and characterized by the use of ^1^H- and ^13^C-NMR (Bruker AVANCE III HD 600, Billerica, MA, USA, deuterated chloroform), IR (IRAffinity-1 FTIR spectrometer; Shimadzu Corporation, Kyoto, Japan, KBr pellet), and MS analyses (Bruker Impact II, Billerica, MA, USA). The following compounds were synthesized: 28-propynoylbetulin (**2**) [25]; 3-diethoxyphosphoryl-28-propynoylbetulin (**3**) [35]; 30-diethoxyphosphoryl-28-propynoylbetulin (**4**) [34]; and 28-diethoxyphosphorylbetulin (**5A**) [36]. Spectroscopic (^1^H, ^13^C, and ^31^P NMR) and melting point data for compounds 2–4 were consistent with the literature values.

Before biological testing, stock solutions were prepared immediately before use by dissolving test substances in dimethyl sulfoxide (DMSO; Sigma Aldrich, Poznań, Polska) at concentration of 20 mM. Working solutions were prepared by the dilution of stock solutions with culture medium. The final concentration range of tested agents was 0.01–30 μM. The final DMSO concentration in culture media was adjusted to 0.2%.

#### Synthesis of 28-diethoxyphosphoryl-3-propynoylbetulin (**5**)

The mixture of 28-diethoxyphosporylbetulin **5A** (0.58 g, 1.0 mmol) and 1.1 mmol of propiolic acid in dichloromethane (3.0 mL) was cooled to −10 °C with an ice/salt bath. Then, a solution of DCC (0.22 g, 1.12 mmol) and DMAP (N,N-dimethylaminopyridine; 0.005 g, 0.04 mmol) in dichloromethane (1.2 mL) was gradually added in three portions (Figure 10). The reaction was carried out under an inert gas (argon) atmosphere for 24 h. The progress of the reaction was monitored by TLC (SiO_2_, hexane/ethyl acetate, 3: 2 *v*/*v*). After completion of the reaction, the volatile components were evaporated under reduced pressure. The residue was purified by column chromatography (SiO_2_, hexane/ethyl acetate, 3: 2 *v*/*v*).

Yield: 36%, m. p. 189–191 °C.

TLC (hexane: ethyl acetate, 3:2, *v*/*v*): *R*f = 0.17.

^1^H-NMR (600 MHz, CDCl_3_) δ (ppm): 0.81 (m, 1H, H-5), 0.87 (s, 3H, CH_3_), 0.89 (s, 3H, CH_3_), 0.91 (s, 3H, CH_3_), 1.01 (s, 3H, CH_3_), 1.05 (s, 3H, CH_3_), 1.39 (m, 6H, 2x OCH_2_CH_3_), 1.70 (s, 3H, CH_3_), 2.38 (m, 1H, H-19), 2.88 (s, 1H, C≡CH), 3.77 (dd, J = 10.8, 1H, H-28), 4.14 (m, 4H, 2x OCH_2_CH_3_), 4.21 (dd, J = 10.8, 1H, H-28), 4.61 (d, J= 1.8 Hz, 1H, H-29), 4.62 (m, 1H, H-3), 4.70 (d, J= 1.8 Hz, 1H, H-29).

^13^C-NMR (150 MHz, CDCl_3_) δ (ppm): 14.7; 16.0; 16.1; 16.2 (OCH_2_CH_3_); 16.5; 18.1; 19.1; 20.8; 23.5; 25.1; 26.9; 27.9; 29.2; 29.5; 34.1; 37.0; 37.6; 37.9; 38.3; 40.9; 42.7; 47.2; 47.7; 48.6; 50.2; 55.4; 63.7 (OCH_2_CH_3_); 66.0 (d, JCP=6 Hz, C-28)); 74.1 (HC≡C); 75.1 (HC≡C); 83.7; 109.9; 150.0; 152.8 (C(O)).

^31^P NMR (243 MHz, CDCl_3_) δ (ppm): −0.16.

IR (KBr, cm^−1^) ν: 2103 (C≡CH), 1718 (C=O), 1267 (P=O), 1025 (P-O-C).

HR MS (APCI) m/z: C_37_H_58_O_6_P [(M-H)-], Calc. 629.3971; Found 629.3964.

### 4.2. Cell Cultures

Human breast cancer cell lines used in this research were purchased from the American Type Culture Collection. MCF-7 cells (ATCC^®^ HTB-22™) were cultured in EMEM (Eagle’s Minimum Essential Medium; Sigma Aldrich, Poznań, Polska) supplemented with 10% fetal bovine serum, 20 mM HEPES (4-(2-hydroxyethyl)-1-piperazineethanesulfonic acid; Thermo Fisher Scientific, Waltham, MA, USA), 0.01 mg/mL insulin, 1 mM sodium pyruvate, 1× MEM-non essential amino acids, 100 U/mL penicillin, and 100 μg/mL streptomycin (all from Sigma Aldrich, Poznań, Polska). The SK-BR-3 (ATCC^®^ HTB-30™) cell line was grown in McCoy’s 5a medium (Sigma Aldrich, Poznań, Polska) containing 10% fetal bovine serum, 20 mM HEPES, 100 U/mL penicillin, and 100 μg/mL streptomycin. The cells were maintained at 37 °C in a humidified atmosphere containing 5% CO_2_.

### 4.3. Sulforhodamine B (SRB) Assay

In Vitro Toxicology Assay Kit, Sulforhodamine B Based (Sigma-Aldrich, Poznań, Polska) was used to evaluate cellular growth. Cells were seeded in 96-well plates at an initial density of 3 × 10^3^ cells per well in 200 µL of culture medium. Cells were allowed to attach and grow for 1 day prior to the exposure to test reagents. Then, the media were replaced with the working solutions, and cells were exposed to the tested substances for 72 h. At the end of the incubation period, culture media were aspirated, and cells were fixed at 4 °C using 10% trichloroacetic acid solution, washed two times with deionized water, and eventually stained with 0.4% sulforhodamine B (in 1.0% acetic acid). After washing out the unincorporated stain using 1.0% acetic acid solution, the bound dye was solubilized in 200 μL of 10 mM tris(hydroxymethyl)aminomethane solution. Absorbance was measured at 570 nm and 690 nm (reference wavelength) using the MRX Revelation plate reader (Dynex Technologies, Chantilly, VA, USA).

### 4.4. Determination of DNA Synthesis

The rate of DNA synthesis was determined by the bromodeoxyuridine (BrdU) incorporation assay with the use of the Cell Proliferation ELISA, BrdU (colorimetric) kit (Roche, Basel, Switzerland). Cells were plated in 96-well plates at a density of 5 × 10^3^ cells per well in 100 µL of culture medium and cultured for 24 h to allow cell adhesion. Then, the media were replaced with the working solutions of tested substances, and cells were incubated for the next 24 h. BrdU (final concentration 10 µM) was added to culture media after 12 h of incubation with tested substances, and incubation was continued for the next 12 h. Then, media were removed, and 200 µL of FixDenat was added to the wells to fix the cells and denature DNA. The amount of BrdU retained in cells was determined immunoenzymatically according to producer’s instruction. Absorbance was measured at 450 nm and 690 nm (reference wavelength).

### 4.5. Cytotoxicity Assays

Release of lactate dehydrogenase (LDH) was determined to evaluate cellular death by means of the In Vitro Toxicology Assay Kit, Lactate Dehydrogenase Based (Sigma Aldrich, Poznań, Polska). Cells were plated and cultured as described above for DNA synthesis assay. To some wells, culture media without cells were added (background control). In selected wells of each plate, cells were lysed prior to the assay to determine maximal LDH release (high control). Wells with untreated cells were used as the low control of the assay. LDH activity in media was assessed according to the manufacturer’s instruction. The absorbance was measured at wavelengths of 490 nm and 690 nm (background) and the percentage of liberated enzyme was calculated for cells growing in the presence of betulin derivatives according to the formula:

Cytotoxicity [%] = (A_T_ − A_L_/A_H_ − A_L_) ∗ 100

where A_T_—absorbance of treated wells; A_L_—low control; A_H_—high control (maximal LDH release).

Dead cells were visualized using the Live/Dead Viability/Cytotoxicity Kit (Thermo Fisher Scientific, Waltham, MA, USA) containing two fluorescent dyes: calcein AM, which produces green fluorescence in living cells (after conversion to polyanionic calcein inside the cell) and ethidium homodimer-1 conferring red fluorescence to dead cells. Cells were cultured in 24-well tissue culture plates, treated with the compound **4** for 24 h, stained according to the manufacturer’s protocol, observed under a fluorescence microscope (Nikon Eclipse TS-100F, NIKON, Tokyo, Japan), and photographed using a Nikon DS-Fi1 digital camera.

### 4.6. DNA Fragmentation Assay

Internucleosomal fragmentation of genomic DNA was determined by means of the Cell Death Detection ELISA^PLUS^ kit (Roche, Basel, Switzerland). This method relies on immunoenzymatic detection of DNA fragments in the cytoplasmic cell fraction. Cells were plated and cultured as described above for DNA synthesis assay. At the end of the 24 h incubation period, plates were centrifuged (200× *g*; 10 min), media were removed, and cells were lysed for 30 min at room temperature. Then, lysates were spun (200× *g*; 10 min) and 20 µL of supernatant (cytoplasmic fraction) was moved to the streptavidin-coated 96-well plate. The sandwich ELISA assay was carried out according to the manufacturer’s protocol. Absorbance was measured at 405 nm and 490 nm (reference wavelength) and enrichment of mono and oligonucleosomes released into the cytoplasm was calculated:

EF = A_S_/A_C_ (1)

where A_S_ is the absorbance of the sample, A_C_ is the absorbance of control, and EF is the enrichment factor.

### 4.7. Gene and Protein Expression

Cells were plated into tissue culture dishes (21.5 cm^2^) at an initial density of 1 × 10^6^ cells/dish. Cells were allowed to attach and grow for 72 h prior to exposure to the test substance. Total RNA was isolated using the GeneMATRIX Universal Purification Kit (EUR_X_) together with the on-column DNase digestion. The RNA concentration was assessed fluorometrically by means of the Quant-iT™ RiboGreen^®^ RNA Assay Kit (Thermo Fisher Scientific, Waltham, MA, USA). The reverse transcription and amplification reactions were done on 10 ng of RNA, in a single step reaction with specific primers, using the TaqMan^®^ RNA-to-CT™ 1-Step Kit (Thermo Fisher Scientific, Waltham, MA, USA). Analysis of mRNA expression levels for p21^WAF1/Cip1^ was done using a commercially available, pre-validated set of primers and fluorescent, FAM (6-carboxyfluorescein)-labeled probe, purchased from Thermo Fisher Scientific (Waltham, MA, USA): Hs99999142_m1. Expression levels were normalized with TBP (Hs99999910_m1) as a reference gene. Amplification was performed using the CFX Connect Real-Time PCR Detection System (Bio-Rad, Hercules, CA, USA).

The level of p21^WAF1/Cip1^ protein in cell lysates was determined by means of the ELISA technique. At the end of incubation with the selected betulin derivative, cells were washed with cold PBS (phosphate buffered saline, pH 7.4), scraped, and centrifuged (400× *g*; 10 min; 4 °C). The pellet was lysed on ice in the cell extraction buffer supplemented with protease inhibitor cocktail and PMSF (phenylmethylsulfonyl fluoride) (both from Sigma-Aldrich, Poznań, Polska). Lysates were clarified by centrifugation (13,000 rpm; 10 min; 4 °C) and stored at −80 °C until analysis. Concentration of the p21^WAF1/Cip1^ protein was determined using the p21^WAF1/Cip1^ Total ELISA Kit (Thermo Fisher Scientific, Waltham, MA, USA). Results were normalized relative to the total cellular protein content, which was determined using Bradford method [74].

### 4.8. Assessment of Mitochondrial Membrane Potential

To visualize changes in the transmembrane potential of mitochondria using fluorescence microscopy, cells were seeded into 24-well tissue culture plates with glass bottoms (Wuxi NEST Biotechnology) at an initial density of 10^5^ cells/well in 1 mL of the appropriate medium. Cells were allowed to attach and grow for 48 h prior to treatment. Then, culture media were replaced with the working solutions of tested compounds. At selected time points, tetramethylrhodamine methyl ester (TMRM; Sigma Aldrich, Poznań, Polska), a fluorescent cationic dye accumulating in mitochondria in a potential-dependent manner, was added to culture media (final concentration 100 nM), and plates were placed in the CO_2_ incubator for 30 min. Stained cells were observed under the fluorescence microscope and photographed. For cytometric analysis, cells were seeded into tissue culture dishes and cultured for 48 h prior to treatment. After treatment for 3 h and 6 h, cells were incubated with TMRM, detached using trypsin/EDTA (ethylenediamine tetraacetic acid) solution, resuspended in PBS, and analyzed using a flow cytometer (FACS AriaII, Becton Dickinson, San Jose, CA, USA).

### 4.9. Caspase-3 Activity Assay

Activity of caspase-3 was measured using the Colorimetric Caspase-3 Assay Kit (Sigma-Aldrich, Poznań, Polska). Cell culture and treatment were done as described in the preceding section (Gene and Protein Expression). Cells were incubated with betulin derivative for 6 h and then scraped on ice in culture medium, pelleted by centrifugation (400× *g*; 10 min; 4 °C), and washed with ice-cold PBS. Subsequently, cells were lysed by incubation in lysis buffer on ice for 20 min. Lysates were clarified by centrifugation (16,000× *g*; 10 min; 4 °C) and stored at −80 °C until assayed. Enzyme activity was determined in 96-well plates using Ac-DEVD-pNA as substrate. Plates with reaction mixtures were incubated at 37 °C, and the amount of p-nitroaniline (pNA) liberated was determined spectrophotometrically at 405 nm. A specific caspase-3 inhibitor (Ac-DEVD-CHO) was added to the reaction mixture in separate control wells to reveal a non-specific cleavage of the substrate. Differences of absorbance between inhibited and uninhibited wells (for each cell lysate) were calculated, and pNA concentrations were obtained from a standard curve. Caspase-3 activity was expressed relative to cellular protein content, which was determined using the Bradford method [74]. Dying cells, with active caspases, were visualized using an Image-iT™ LIVE Red Poly Caspases Detection Kit (Thermo Fisher Scientific, Waltham, MA, USA). This method uses a broad-spectrum fluorescent caspase inhibitor (SR-VAD-FMK) for the detection of activated cascade of caspases. Plasma membrane integrity and nuclear morphology were analyzed using SYTOX Green, a live cell impermeable nucleic acid stain. Then, 24-well plates with stained cells were centrifuged (200× *g*; 10 min), staining solutions were removed, and cells were washed with PBS. Then, plates were centrifuged again, PBS was removed, and cells were fed with 1 mL of fresh medium. Then, cells were observed under the fluorescence microscope and photographed.

### 4.10. Measurement of the Production of Reactive Oxygen Species

To measure generalized intracellular oxidative stress, 2′,7′-dichlorofluorescin diacetate, a cell permeable fluorescent probe (DCFDA; Sigma Aldrich, Poznań, Polska), was used [75]. Cells were plated into black 96-well microplates (5 × 10^3^ cells/well) and cultured in standard conditions for 48 h. Subsequently, media were removed, cells were washed with HBSS (Hank’s Balanced Salt Solution) and incubated in 100 μL of DCFDA solution (100 µM in HBSS) for 30 min, in the CO_2_ incubator. Then, cells were washed with HBSS and treated with the tested substance dissolved in HBSS (100 µL/well) for up to 60 min. Fluorescence emission was measured at a wavelength of 535 nm after excitation at 485 nm using the Triad LT Multimode Reader (Dynex Technologies, Chantilly, VA, USA).

### 4.11. Acridine Orange Staining

Vital staining of cells with acridine orange was used for visualization of the morphological changes of cell nuclei. In particular, the condensation and marginalization of chromatin as well as fragmentation of nuclei, typical manifestations of apoptosis, were looked for. Cell cultures were prepared as described above for TMRM staining. At selected time points, cells were stained with acridine orange (final concentration 5 μg/mL), observed under the fluorescence microscope, and photographed.

### 4.12. Statistical Analysis

PCR efficiency and Cq values were determined by means of LinRegPCR software [76]. Calculations of relative gene expression levels as well as statistical analysis were done using the REST 2009 [77]. All the remaining results were analyzed using a one-way ANOVA followed by a Tukey post hoc test. When the assumptions of ANOVA were not met, a nonparametric Kruskal–Wallis test was applied. A *p* value of <0.05 was considered statistically significant. Analysis was performed using Statistica 13.1 software (StatSoft, Tulsa, Okay, USA). IC_50_ values were calculated using GraphPad Prism 8 (GraphPad Software Inc., San Diego, CA, USA).

## 5. Conclusions

In conclusion, 30-diethoxyphosphoryl-28-propynoylbetulin proved to be a potent cytotoxic agent against SK-BR-3 and MCF-7 breast cancer cell lines. Inhibition of cell growth was found to be associated with an up-regulation of p21^WAF1/Cip1^ gene expression. Decrease in cell viability was preceded by alterations of the ROS level and loss of the mitochondrial membrane potential. DNA fragmentation and activation of caspase-3 (in SK-BR-3 cells) indicated the possibility of triggering apoptotic mechanisms. However, cells treated with compound **4** failed to display apoptotic nuclear morphology, which was represented by chromatin condensation and karyorrhexis. The necrotic-like cell death mode could possibly result from the fast disturbance of mitochondrial function and deficiency of energy. Cell death through necrosis is believed to be more immunogenic than cell demise through apoptosis [78]. It leads to the release of inflammatory stimuli and recruitment of a distinct set of immune cells. Therefore, the mode of cancer cell killing is a factor of big importance for the breaking of immune tolerance to tumor antigens and, as a consequence, the effectiveness of therapy.

## Figures and Tables

**Table 1:** Growth inhibition efficiency (IC_50_) of betulin (**1**) and its derivatives on two breast cancer cell lines after treatment for 72h; table shows arithmetic means ± SD.

Compound	IC_50_ (µM)	
	SK-BR-3	MCF7
**1**	7.93 ± 1.05	13.26 ± 0.75
**2**	3.83 ± 0.34	n.d.
**3**	2.89 ± 0.56	n.d.
**4**	2.12 ± 0.59	5.34 ± 0.03
**5**	3.49 ± 0.32	12.88 ± 0.96

## Data Availability

The data presented in this study are available on request from the corresponding author.

## Supplementary Materials

The following are available online, Figures S1–S3: Nuclear Magnetic Resonance spectra for compound 5 (Figure S1. ^1^H NMR (600 MHz, CDCl_3_; Figure S2. ^13^C NMR (243 MHz, CDCl_3_; Figure S3. ^31^P NMR, (150 MHz, CDCl_3_); Figure S4: IR spectrum for compound 5 (KBr pellet); Figure S5: HR MS, compound 5.

## Sample Availability

Samples of the compounds are not available from the authors.

## References

[1] Rzeski W., Stepulak A., Szymanski M., Juszczak M., Grabarska A., Sifringer M., Kaczor J., Kandefer-Szerszen M. (2009). Betulin elicits anti-cancer effects in tumour primary cultures and cell lines in vitro. Basic Clin. Pharmacol. Toxicol..

[2] Alakurtti S., Makela T., Koskimies S., Yli-Kauhaluoma J. (2006). Pharmacological properties of the ubiquitous natural product betulin. Eur. J. Pharm. Sci..

[3] Li Y., He K., Huang Y., Zheng D., Gao C., Cui L., Jin Y.H. (2010). Betulin induces mitochondrial cytochrome c release associated apoptosis in human cancer cells. Mol. Carcinog..

[4] Orchel A., Kulczycka A., Chodurek E., Bebenek E., Borkowska P., Boryczka S., Kowalski J., Dzierzewicz Z. (2014). Influence of betulin and 28-O-propynoylbetulin on proliferation and apoptosis of human melanoma cells (G-361). Postepy Hig Med. Dosw (Online).

[5] Pyo J.S., Roh S.H., Kim D.K., Lee J.G., Lee Y.Y., Hong S.S., Kwon S.W., Park J.H. (2009). Anti-cancer effect of Betulin on a human lung cancer cell line: A pharmacoproteomic approach using 2 D SDS PAGE coupled with nano-HPLC tandem Mass Spectrometry. Planta Med..

[6] Zhou Z., Zhu C., Cai Z., Zhao F., He L., Lou X., Qi X. (2018). Betulin induces cytochrome c release and apoptosis in colon cancer cells via NOXA. Oncol. Lett..

[7] Fulda S., Kroemer G. (2009). Targeting mitochondrial apoptosis by betulinic acid in human cancers. Drug Discov Today.

[8] Zeng A.Q., Yu Y., Yao Y.Q., Yang F.F., Liao M., Song L.J., Li Y.L., Yu Y., Li Y.J., Deng Y.L. (2018). Betulinic acid impairs metastasis and reduces immunosuppressive cells in breast cancer models. Oncotarget.

[9] Fulda S., Friesen C., Los M., Scaffidi C., Mier W., Benedict M., Nunez G., Krammer P.H., Peter M.E., Debatin K.M. (1997). Betulinic acid triggers CD95 (APO-1/Fas)- and p53-independent apoptosis via activation of caspases in neuroectodermal tumors. Cancer Res..

[10] Mullauer F.B., Kessler J.H., Medema J.P. (2009). Betulin is a potent anti-tumor agent that is enhanced by cholesterol. PLoS ONE.

[11] Fulda S. (2009). Tumor resistance to apoptosis. Int. J. Cancer.

[12] Elmore S. (2007). Apoptosis: A review of programmed cell death. Toxicol. Pathol..

[13] Zeiss C.J. (2003). The apoptosis-necrosis continuum: Insights from genetically altered mice. Vet. Pathol..

[14] Slee E.A., Harte M.T., Kluck R.M., Wolf B.B., Casiano C.A., Newmeyer D.D., Wang H.G., Reed J.C., Nicholson D.W., Alnemri E.S. (1999). Ordering the cytochrome c-initiated caspase cascade: Hierarchical activation of caspases-2, -3, -6, -7, -8, and -10 in a caspase-9-dependent manner. J. Cell Biol..

[15] Wang J., Guo W., Zhou H., Luo N., Nie C., Zhao X., Yuan Z., Liu X., Wei Y. (2015). Mitochondrial p53 phosphorylation induces Bak-mediated and caspase-independent cell death. Oncotarget.

[16] Vanden Berghe T., Linkermann A., Jouan-Lanhouet S., Walczak H., Vandenabeele P. (2014). Regulated necrosis: The expanding network of non-apoptotic cell death pathways. Nat. Rev. Mol. Cell Biol..

[17] Leist M., Single B., Castoldi A.F., Kuhnle S., Nicotera P. (1997). Intracellular adenosine triphosphate (ATP) concentration: A switch in the decision between apoptosis and necrosis. J. Exp. Med..

[18] Garcia-Belinchon M., Sanchez-Osuna M., Martinez-Escardo L., Granados-Colomina C., Pascual-Guiral S., Iglesias-Guimarais V., Casanelles E., Ribas J., Yuste V.J. (2015). An Early and Robust Activation of Caspases Heads Cells for a Regulated Form of Necrotic-like Cell Death. J. Biol. Chem..

[19] Mullauer F.B., Kessler J.H., Medema J.P. (2009). Betulinic acid induces cytochrome c release and apoptosis in a Bax/Bak-independent, permeability transition pore dependent fashion. Apoptosis.

[20] Potze L., Mullauer F.B., Colak S., Kessler J.H., Medema J.P. (2014). Betulinic acid-induced mitochondria-dependent cell death is counterbalanced by an autophagic salvage response. Cell Death Dis..

[21] Kessler J.H., Mullauer F.B., de Roo G.M., Medema J.P. (2007). Broad in vitro efficacy of plant-derived betulinic acid against cell lines derived from the most prevalent human cancer types. Cancer Lett..

[22] Gauthier C., Legault J., Lavoie S., Rondeau S., Tremblay S., Pichette A. (2009). Synthesis and cytotoxicity of bidesmosidic betulin and betulinic acid saponins. J. Nat. Prod..

[23] Rajendran P., Jaggi M., Singh M.K., Mukherjee R., Burman A.C. (2008). Pharmacological evaluation of C-3 modified Betulinic acid derivatives with potent anticancer activity. Invest. New Drugs.

[24] Drag-Zalesinska M., Drag M., Poreba M., Borska S., Kulbacka J., Saczko J. (2017). Anticancer properties of ester derivatives of betulin in human metastatic melanoma cells (Me-45). Cancer Cell Int..

[25] Boryczka S., Bebenek E., Wietrzyk J., Kempinska K., Jastrzebska M., Kusz J., Nowak M. (2013). Synthesis, structure and cytotoxic activity of new acetylenic derivatives of betulin. Molecules.

[26] Chrobak E., Bebenek E., Kadela-Tomanek M., Latocha M., Jelsch C., Wenger E., Boryczka S. (2016). Betulin Phosphonates; Synthesis, Structure, and Cytotoxic Activity. Molecules.

[27] Yan X., Yang L., Feng G., Yu Z., Xiao M., Cai W., Xing Y., Bai S., Guo J., Wang Z. (2018). Lup-20(29)-en-3beta,28-di-yl-nitrooxy acetate affects MCF-7 proliferation through the crosstalk between apoptosis and autophagy in mitochondria. Cell Death Dis..

[28] Chakraborty B., Dutta D., Mukherjee S., Das S., Maiti N.C., Das P., Chowdhury C. (2015). Synthesis and biological evaluation of a novel betulinic acid derivative as an inducer of apoptosis in human colon carcinoma cells (HT-29). Eur. J. Med. Chem..

[29] Szoka L., Karna E., Hlebowicz-Sarat K., Karaszewski J., Boryczka S., Palka J.A. (2017). Acetylenic derivative of betulin induces apoptosis in endometrial adenocarcinoma cell line. Biomed. Pharmacother..

[30] Bebenek E., Kadela-Tomanek M., Chrobak E., Wietrzyk J., Sadowska J., Boryczka S. (2017). New acetylenic derivatives of betulin and betulone, synthesis and cytotoxic activity. Med. Chem. Res..

[31] Zaklos-Szyda M., Pawlik N., Polka D., Nowak A., Koziolkiewicz M., Podsedek A. (2019). Viburnum opulus Fruit Phenolic Compounds as Cytoprotective Agents Able to Decrease Free Fatty Acids and Glucose Uptake by Caco-2 Cells. Antioxidants (Basel).

[32] Huang L., Mackenzie G.G., Sun Y., Ouyang N., Xie G., Vrankova K., Komninou D., Rigas B. (2011). Chemotherapeutic properties of phospho-nonsteroidal anti-inflammatory drugs, a new class of anticancer compounds. Cancer Res..

[33] Rosłon M., Jastrzębska A., Sitarz K., Książek I., Koronkiewicz M., Anuszewska E., Jaworska M., Dudkiewicz-Wilczyńska J., Ziemkowska W., Basiak D. (2019). The toxicity in vitro of titanium dioxide nanoparticles modified with noble metals on mammalian cells. Int. J. Appl. Ceram. Technol..

[34] Boryczka S., Chrobak E., Szymura A., Latocha M., Kadela M., Bębenek E. (2017). Acetylenowe pochodne 30-fosforanu betuliny o działaniu przeciwnowotworowym, sposób ich wytwarzania i zastosowanie (Antitumor acetylenic derivatives of betulin 30-phosphate, method of their preparation and application). RP Patent.

[35] Chrobak E., Kadela-Tomanek M., Bebenek E., Marciniec K., Wietrzyk J., Trynda J., Pawelczak B., Kusz J., Kasperczyk J., Chodurek E. (2019). New phosphate derivatives of betulin as anticancer agents: Synthesis, crystal structure, and molecular docking study. Bioorg. Chem..

[36] Sachet M., Liang Y.Y., Oehler R. (2017). The immune response to secondary necrotic cells. Apoptosis.

[37] Bray F., Ferlay J., Soerjomataram I., Siegel R.L., Torre L.A., Jemal A. (2018). Global cancer statistics 2018: GLOBOCAN estimates of incidence and mortality worldwide for 36 cancers in 185 countries. CA Cancer J. Clin..

[38] Ferlay J., Colombet M., Soerjomataram I., Mathers C., Parkin D.M., Pineros M., Znaor A., Bray F. (2018). Estimating the global cancer incidence and mortality in 2018: GLOBOCAN sources and methods. Int. J. Cancer.

[39] Barnard M.E., Boeke C.E., Tamimi R.M. (2015). Established breast cancer risk factors and risk of intrinsic tumor subtypes. Biochim. Biophys. Acta.

[40] Nelson H.D., Zakher B., Cantor A., Fu R., Griffin J., O’Meara E.S., Buist D.S., Kerlikowske K., van Ravesteyn N.T., Trentham-Dietz A. (2012). Risk factors for breast cancer for women aged 40 to 49 years: A systematic review and meta-analysis. Ann. Intern. Med..

[41] ACS (2017). Breast Cancer Facts & Figures 2017–2018.

[42] ACS (2018). Cancer Facts & Figures 2018.

[43] Tamimi R.M., Colditz G.A., Hazra A., Baer H.J., Hankinson S.E., Rosner B., Marotti J., Connolly J.L., Schnitt S.J., Collins L.C. (2012). Traditional breast cancer risk factors in relation to molecular subtypes of breast cancer. Breast Cancer Res. Treat..

[44] Oakes S.R., Vaillant F., Lim E., Lee L., Breslin K., Feleppa F., Deb S., Ritchie M.E., Takano E., Ward T. (2012). Sensitization of BCL-2-expressing breast tumors to chemotherapy by the BH3 mimetic ABT-737. Proc. Natl. Acad. Sci. USA.

[45] Booy E.P., Henson E.S., Gibson S.B. (2011). Epidermal growth factor regulates Mcl-1 expression through the MAPK-Elk-1 signalling pathway contributing to cell survival in breast cancer. Oncogene.

[46] Zhuo Z.J., Xiao M.J., Lin H.R., Luo J., Wang T. (2018). Novel betulin derivative induces anti-proliferative activity by G2/M phase cell cycle arrest and apoptosis in Huh7 cells. Oncol. Lett..

[47] Drag-Zalesinska M., Wysocka T., Borska S., Drag M., Poreba M., Choromanska A., Kulbacka J., Saczko J. (2015). The new esters derivatives of betulin and betulinic acid in epidermoid squamous carcinoma treatment—In vitro studies. Biomed. Pharmacother..

[48] Majeed R., Hamid A., Sangwan P.L., Chinthakindi P.K., Koul S., Rayees S., Singh G., Mondhe D.M., Mintoo M.J., Singh S.K. (2014). Inhibition of phosphotidylinositol-3 kinase pathway by a novel naphthol derivative of betulinic acid induces cell cycle arrest and apoptosis in cancer cells of different origin. Cell Death Dis..

[49] Bebenek E., Jastrzebska M., Kadela-Tomanek M., Chrobak E., Orzechowska B., Zwolinska K., Latocha M., Mertas A., Czuba Z., Boryczka S. (2017). Novel Triazole Hybrids of Betulin: Synthesis and Biological Activity Profile. Molecules.

[50] Rello S., Stockert J.C., Moreno V., Gamez A., Pacheco M., Juarranz A., Canete M., Villanueva A. (2005). Morphological criteria to distinguish cell death induced by apoptotic and necrotic treatments. Apoptosis.

[51] Soriano J., Mora-Espi I., Alea-Reyes M.E., Perez-Garcia L., Barrios L., Ibanez E., Nogues C. (2017). Cell Death Mechanisms in Tumoral and Non-Tumoral Human Cell Lines Triggered by Photodynamic Treatments: Apoptosis, Necrosis and Parthanatos. Sci. Rep..

[52] Ormerod M.G., Sun X.M., Brown D., Snowden R.T., Cohen G.M. (1993). Quantification of apoptosis and necrosis by flow cytometry. Acta Oncol..

[53] Gukovskaya A.S., Perkins P., Zaninovic V., Sandoval D., Rutherford R., Fitzsimmons T., Pandol S.J., Poucell-Hatton S. (1996). Mechanisms of cell death after pancreatic duct obstruction in the opossum and the rat. Gastroenterology.

[54] Ankarcrona M., Dypbukt J.M., Bonfoco E., Zhivotovsky B., Orrenius S., Lipton S.A., Nicotera P. (1995). Glutamate-induced neuronal death: A succession of necrosis or apoptosis depending on mitochondrial function. Neuron.

[55] Dacheux D., Toussaint B., Richard M., Brochier G., Croize J., Attree I. (2000). Pseudomonas aeruginosa cystic fibrosis isolates induce rapid, type III secretion-dependent, but ExoU-independent, oncosis of macrophages and polymorphonuclear neutrophils. Infect. Immun..

[56] Hirsch T., Marchetti P., Susin S.A., Dallaporta B., Zamzami N., Marzo I., Geuskens M., Kroemer G. (1997). The apoptosis-necrosis paradox. Apoptogenic proteases activated after mitochondrial permeability transition determine the mode of cell death. Oncogene.

[57] Lemasters J.J., Qian T., Bradham C.A., Brenner D.A., Cascio W.E., Trost L.C., Nishimura Y., Nieminen A.L., Herman B. (1999). Mitochondrial dysfunction in the pathogenesis of necrotic and apoptotic cell death. J. Bioenerg. Biomembr..

[58] Eguchi Y., Shimizu S., Tsujimoto Y. (1997). Intracellular ATP levels determine cell death fate by apoptosis or necrosis. Cancer Res..

[59] Eguchi Y., Srinivasan A., Tomaselli K.J., Shimizu S., Tsujimoto Y. (1999). ATP-dependent steps in apoptotic signal transduction. Cancer Res..

[60] Kushnareva Y., Newmeyer D.D. (2010). Bioenergetics and cell death. Ann. N. Y. Acad. Sci..

[61] Robertson J.D., Orrenius S., Zhivotovsky B. (2000). Review: Nuclear events in apoptosis. J. Struct. Biol..

[62] Oppenheim R.W., Flavell R.A., Vinsant S., Prevette D., Kuan C.Y., Rakic P. (2001). Programmed cell death of developing mammalian neurons after genetic deletion of caspases. J. Neurosci..

[63] Zheng T.S., Flavell R.A. (2000). Divinations and surprises: Genetic analysis of caspase function in mice. Exp. Cell Res..

[64] Wen Y., Chen Z., Lu J., Ables E., Scemama J.L., Yang L.V., Lu J.Q., Hu X.H. (2017). Quantitative analysis and comparison of 3D morphology between viable and apoptotic MCF-7 breast cancer cells and characterization of nuclear fragmentation. PLoS ONE.

[65] Zhou Y., Bi Y., Yang C., Yang J., Jiang Y., Meng F., Yu B., Khan M., Ma T., Yang H. (2013). Magnolol induces apoptosis in MCF-7 human breast cancer cells through G2/M phase arrest and caspase-independent pathway. Pharmazie.

[66] Mooney L.M., Al-Sakkaf K.A., Brown B.L., Dobson P.R. (2002). Apoptotic mechanisms in T47D and MCF-7 human breast cancer cells. Br. J. Cancer.

[67] Feinstein-Rotkopf Y., Arama E. (2009). Can’t live without them, can live with them: Roles of caspases during vital cellular processes. Apoptosis.

[68] Miossec C., Dutilleul V., Fassy F., Diu-Hercend A. (1997). Evidence for CPP32 activation in the absence of apoptosis during T lymphocyte stimulation. J. Biol. Chem..

[69] Redza-Dutordoir M., Averill-Bates D.A. (2016). Activation of apoptosis signalling pathways by reactive oxygen species. Biochim. Biophys. Acta.

[70] Burkitt M.J., Wardman P. (2001). Cytochrome C is a potent catalyst of dichlorofluorescin oxidation: Implications for the role of reactive oxygen species in apoptosis. Biochem. Biophys. Res. Commun..

[71] Gupta M.K., Neelakantan T.V., Sanghamitra M., Tyagi R.K., Dinda A., Maulik S., Mukhopadhyay C.K., Goswami S.K. (2006). An assessment of the role of reactive oxygen species and redox signaling in norepinephrine-induced apoptosis and hypertrophy of H9c2 cardiac myoblasts. Antioxid Redox Signal..

[72] Kalota A., Selak M.A., Garcia-Cid L.A., Carroll M. (2015). Eltrombopag modulates reactive oxygen species and decreases acute myeloid leukemia cell survival. PLoS ONE.

[73] Ma E., Jeong S.J., Choi J.S., Nguyen T.H., Jeong C.H., Joo S.H. (2019). MS-5, a Naphthalene Derivative, Induces the Apoptosis of an Ovarian Cancer Cell CAOV-3 by Interfering with the Reactive Oxygen Species Generation. Biomol. Ther. (Seoul).

[74] Bradford M.M. (1976). A rapid and sensitive method for the quantitation of microgram quantities of protein utilizing the principle of protein-dye binding. Anal. Biochem..

[75] Halliwell B., Whiteman M. (2004). Measuring reactive species and oxidative damage in vivo and in cell culture: How should you do it and what do the results mean?. Br. J. Pharmacol..

[76] Ruijter J.M., Ramakers C., Hoogaars W.M., Karlen Y., Bakker O., van den Hoff M.J., Moorman A.F. (2009). Amplification efficiency: Linking baseline and bias in the analysis of quantitative PCR data. Nucleic Acids Res..

[77] Pfaffl M.W., Horgan G.W., Dempfle L. (2002). Relative expression software tool (REST) for group-wise comparison and statistical analysis of relative expression results in real-time PCR. Nucleic Acids Res..

[78] Melcher A., Gough M., Todryk S., Vile R. (1999). Apoptosis or necrosis for tumor immunotherapy: What’s in a name?. J. Mol. Med. (Berl.).

