# Supplementary material for: Anticancer Activity of the Acetylenic Derivative of Betulin Phosphate Involves Induction of Necrotic-Like Death in Breast Cancer Cells In Vitro
Source: Molecules. 2021 Jan 25;26(3):615. doi: 10.3390/molecules26030615 (PMC7865664; doi:10.3390/molecules26030615)
Supplement: Supplementary file 1 [file molecules-26-00615-s001.pdf]

Supplementary materials

Anticancer activity of the acetylenic derivative of betulin phosphate involves induction of necrotic-like death in breast cancer cells *in vitro*

Arkadiusz Orchel[1], Ewa Chodurek[1], Marzena Jaworska-Kik[1], Piotr Paduszyński[1], Anna Kaps[1], Elwira Chrobak[2], Ewa Bębenek[2], Stanisław Boryczka[2], Paulina Borkowska[3], Janusz Kasperczyk[1]*

[1]Department of Biopharmacy, Faculty of Pharmaceutical Sciences in Sosnowiec, Medical University of Silesia, Katowice, Poland; aorchel@sum.edu.pl; echodurek@sum.edu.pl; jkik@sum.edu.pl; ppaduszynski@sum.edu.pl; akaps@sum.edu.pl; janusz.kasperczyk@sum.edu.pl

[2] Department of Organic Chemistry, Faculty of Pharmaceutical Sciences in Sosnowiec, Medical University of Silesia, Katowice, Poland; echrobak@sum.edu.pl; ebebenek@sum.edu.pl; boryczka@sum.edu.pl

[3] Department of Medical Genetics, Faculty of Pharmaceutical Sciences in Sosnowiec, Medical University of Sile-sia, Katowice, Poland; pborkowska@sum.edu.pl

* Correspondence aorchel@sum.edu.pl; Tel.: 48 32 3641247

LIST OF CONTENTS

Nuclear Magnetic Resonance spectra for compound **5** (**Fig. S1-S3**)

Fig. S1. ^{1}H NMR (600 MHz, $CDCl_3$)………………………………………………..………. **3**

Fig. S2. ^{13}C NMR (243 MHz, $CDCl_3$)……………………………………………………. **3**

Fig. S3. ^{31}P NMR, (150 MHz, $CDCl_3$)…………………………………………………… **4**

Fig. S4. IR spectrum for compound **5** (KBr pellet)……………………………….… **4**

Fig. S5. HR MS, compound **5**…………………………………………………….… **5**

Fig. S1. ^{1}H NMR (600 MHz, $CDCl_3$)

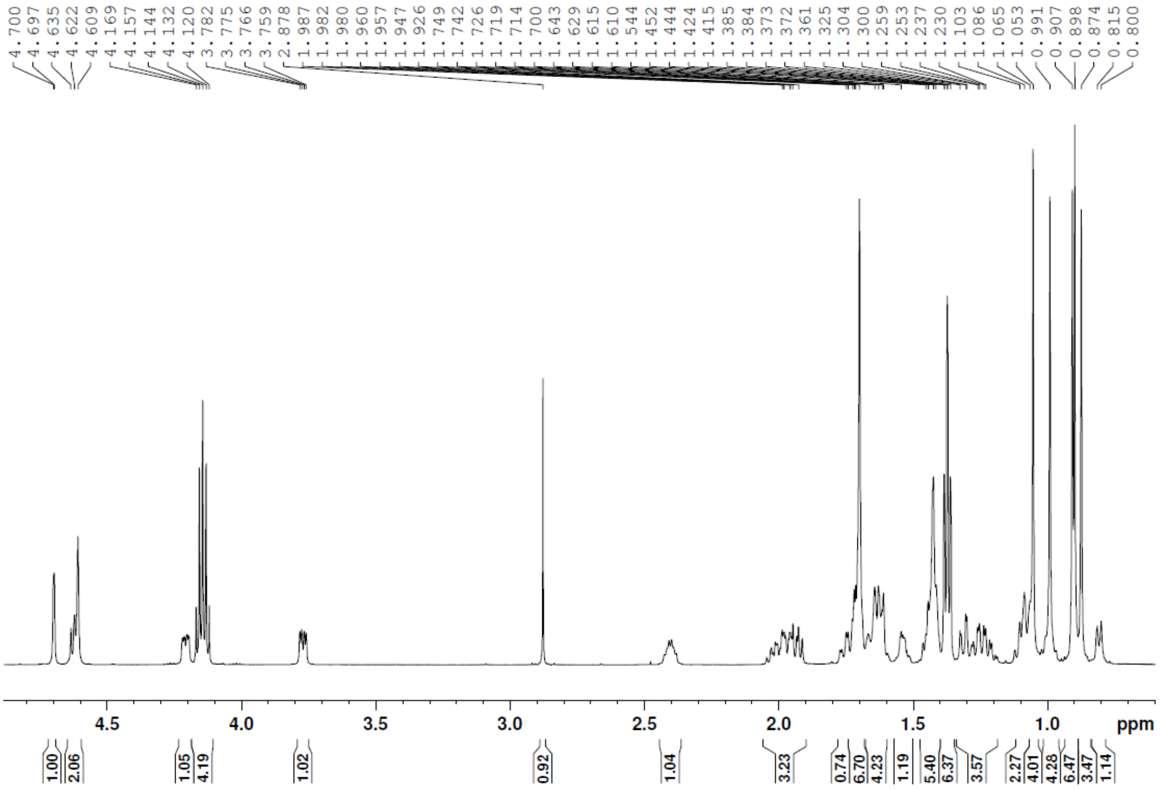

Fig. S2. ^{13}C NMR (243 MHz, $CDCl_3$)

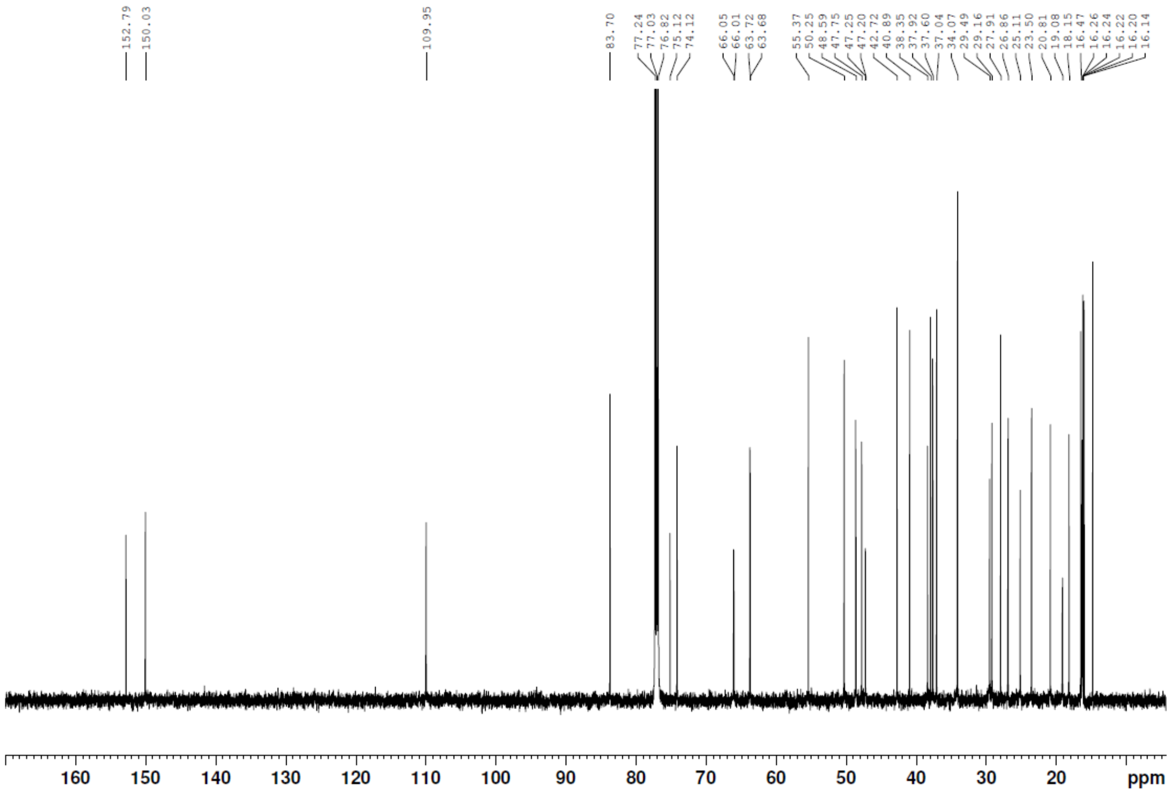

Fig. S3. ^{31}P NMR, (150 MHz, $CDCl_3$)

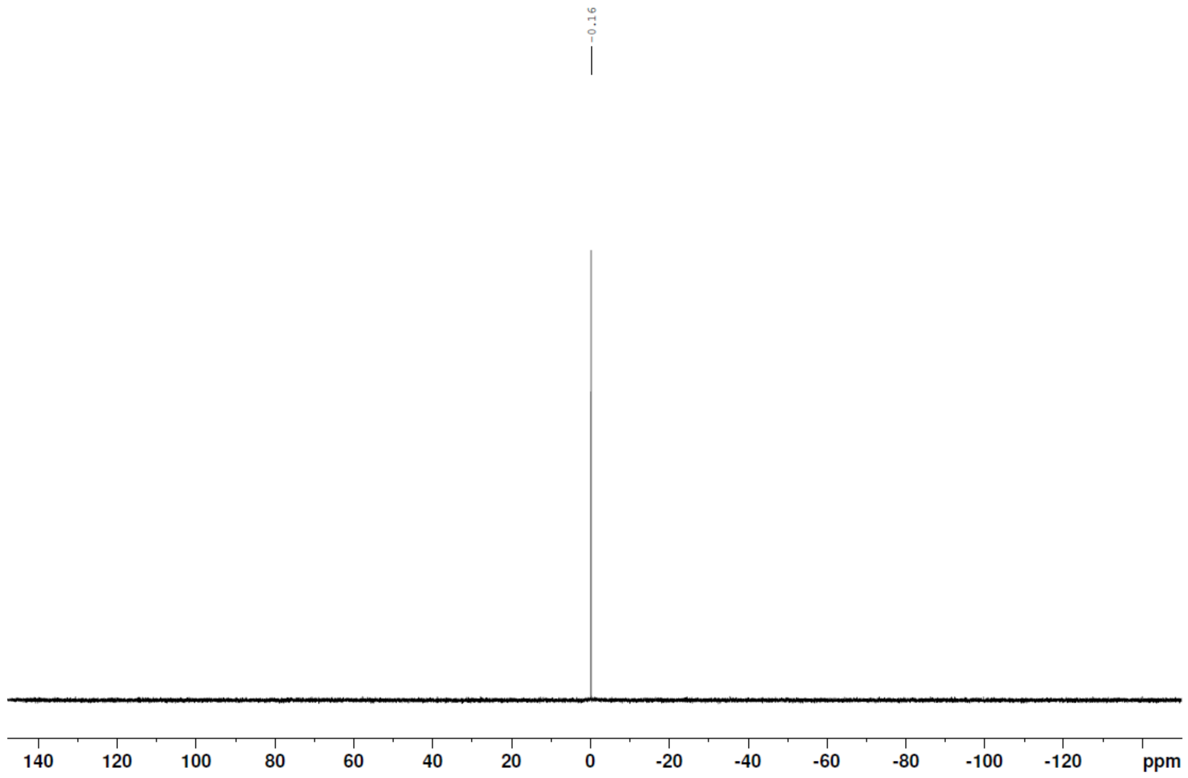

Fig. S4. IR spectrum for compound **5** (KBr pellet)

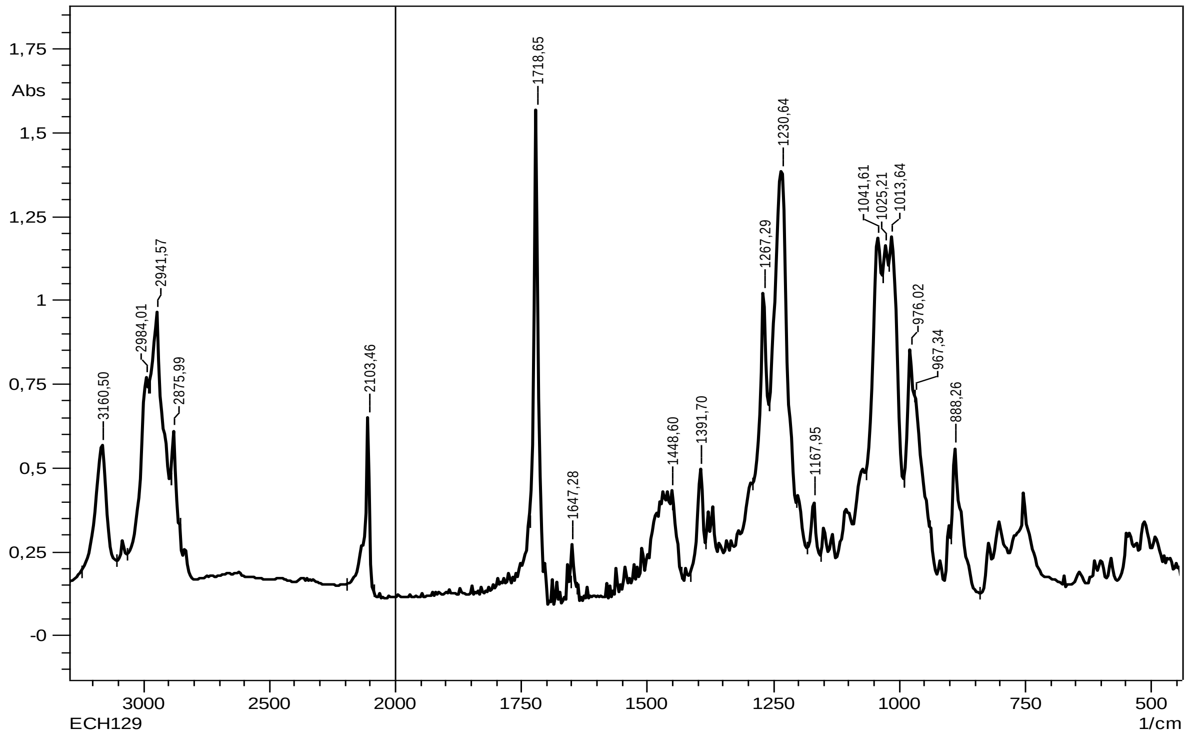

Fig. S5. HR MS, compound **5**

Mass Spectrum List Report

Analysis Info

Analysis Name D:\Data\PM_20171204\ECH 129.d
Method low_mass.m
Sample Name AN 40_18
Comment

Acquisition Date 5/29/2018 11:15:35 AM

Operator KM
Instrument impact II 1825265.10082

Acquisition Parameter

Source Type	APCI	Ion Polarity	Negative	Set Nebulizer	2.0 Bar
Focus	Active	Set Capillary	4000 V	Set Dry Heater	200 °C
Scan Begin	100 m/z	Set End Plate Offset	-500 V	Set Dry Gas	5.0 l/min
Scan End	2000 m/z	Set Charging Voltage	2000 V	Set Divert Valve	Source
		Set Corona	2000 nA	Set APCI Heater	450 °C

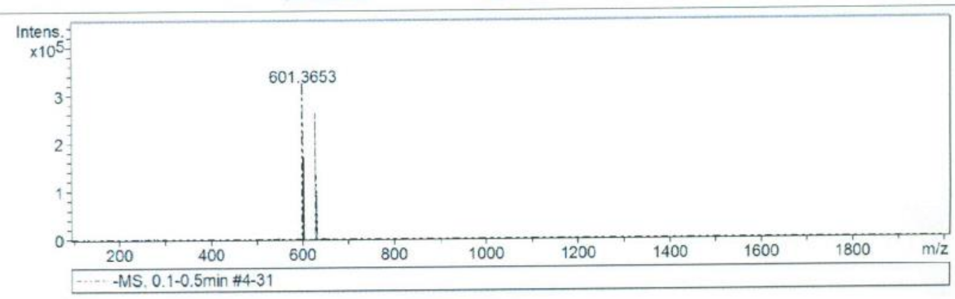

#	m/z	Res.	S/N	I	I %	FWHM
1	601.3653	41998	16780.9	318153	100.0	0.0143
2	629.3964	44274	13738.1	262524	82.5	0.0142
